# Recombinant subunit vaccines for soil-transmitted helminths

**DOI:** 10.1017/S003118201700138X

**Published:** 2017-08-02

**Authors:** JASON B. NOON, RAFFI V. AROIAN

**Affiliations:** Program in Molecular Medicine, University of Massachusetts Medical School, Worcester, MA 01605, USA

**Keywords:** soil-transmitted helminths, anthelmintics, vaccines, adjuvants, protective immunity

## Abstract

Soil-transmitted helminths (STHs) collectively infect one fourth of all human beings, and the majority of livestock in the developing world. These gastrointestinal nematodes are the most important parasites on earth with regard to their prevalence in humans and livestock. Current anthelmintic drugs are losing their efficacies due to increasing drug resistance, particularly in STHs of livestock and drug treatment is often followed by rapid reinfection due to failure of the immune system to develop a protective response. Vaccines against STHs offer what drugs cannot accomplish alone. Because such vaccines would have to be produced on such a large scale, and be cost effective, recombinant subunit vaccines that include a minimum number of proteins produced in relatively simple and inexpensive expression systems are required. Here, we summarize all of the previous studies pertaining to recombinant subunit vaccines for STHs of humans and livestock with the goal of both informing the public of just how critical these parasites are, and to help guide future developments. We also discuss several key areas of vaccine development, which we believe to be critical for developing more potent recombinant subunit vaccines with broad-spectrum protection.

## INTRODUCTION

Helminths (animal parasitic nematodes or roundworms) have debilitated human beings over the course of evolution, whether infecting our livestock, our pets, or us directly. Soil-transmitted helminths (STHs; Table 1) establish chronic infections within the gastrointestinal (GI) tract and are by far the most prevalent. The World Health Organization (WHO) currently estimates that STHs infect 24% of the human population, and infection causes extensive morbidity (Bethony *et al.* 2006). Almost 900 million children are chronically infected, causing stunted growth and delayed cognitive and intellectual development. Millions of pregnant women are also infected, leading to adverse birth outcomes. Although highly concentrated in developing countries of sub-Saharan African, Southeast Asia and tropical regions of Central and South America, those who travel to and from endemic regions are also at risk of getting infected (Bethony *et al.* 2006). Furthermore, when also considering the worldwide prevalence and severity of STHs in livestock (Krecek and Waller, 2006), some of which are known to cause zoonoses (McCarthy and Moore, 2000), greatly reducing the prevalence of these parasites from earth is critical for future global human health and economic growth.

**Table 1.** Biology of soil-transmitted helminth (STH) infections in humans and livestock

STH^a^^,^^b^	Principle host(s)	GI tract location	Ingested host contents	Common sequelae
Ancylostomatidae (hookworms)				
* N. americanus* (*Na*)^c^	Humans	SI	Blood	Blood loss; anaemia; protein malnutrition
* A. duodenale*	Humans	SI	Blood	Blood loss; anaemia; protein malnutrition
* A. ceylanicum* (*Acey*)^c^	Dogs; cats; humans	SI	Blood	Blood loss; anaemia; protein malnutrition
* A. caninum* (*Ac*)	Dogs; cats^d^	SI	Blood	Blood loss; anaemia; protein malnutrition
*Strongyloides* spp.				
* S. stercoralis* (*Ss*)^c^	Humans; dogs; cats	SI	Intestinal fluid	Mostly asymptomatic
* S. fuelleborni*	Humans	SI	Intestinal fluid	Mostly asymptomatic
Trichostrongylidae				
* H. contortus* (*Hc*)^c^	Sheep; goats	Abomasum	Blood	Blood loss; anaemia; oedema
* O. ostertagi* (*Oo*)	Cattle	Abomasum	Abomasal contents	Severe diarrhoea
* T. circumcincta* (*Tci*)	Sheep; goats	Abomasum	Abomasal contents	Severe diarrhoea
Ascaridae (Ascarids)				
* A. lumbricoides*	Humans	SI	Luminal contents	Mostly asymptomatic
* A. suum* (*As*)^c^	Pigs^d^	SI	Luminal contents	Mostly asymptomatic
* T. canis*	Dogs	SI	Luminal contents	Mostly asymptomatic
* T. cati*	Cats	SI	Luminal contents	Mostly asymptomatic
* B. schroederi* (*Bs*)	Giant pandas	SI	Luminal contents	Relatively high mortality rate
* B. procyonis*	Raccoons^d^	SI	Luminal contents	Mostly asymptomatic
Trichurids (whipworms and *Trichinella*)				
* T. trichiura*^c^	Humans	LI	Mucus; blood?	Abdominal pain; bloody diarrhoea
* T. spiralis* (*Ts*)^c^	Pigs; horses; dogs; cats^d^	Muscle; SI	Intestinal fluid	Abdominal pain; diarrhoea

GI, gastrointestinal; SI, small intestine; LI, large intestine.

a Only the STHs that are mentioned in this review and infect humans or livestock (or in the case of *B. schroederi* endangered wild animal species) are listed.

b Only STH species from which the immunogens in Table 2 are derived from given abbreviations in parentheses.

c Genome has been sequenced.

d Relatively frequent reported transmissions from principle host(s) to humans.

**Table 2.** Summary of recombinant subunit vaccine studies for STHs

Immunogen	Adjuvant (animal used)	Significant immune responses	Vaccine efficacy (significant findings)^a^	Reference
r*Ac*-MTP-1	AS02 (beagles) Quil-A (hamsters)	IgG2 Total IgG	FECs and burdens negatively correlated with IgG2 43% lower FECs	Hotez *et al.* (2003) Mendez *et al.* (2005)
r*Ac*-ASP-2	AS03 (beagles) Quil-A (hamsters)	IgG1; IgG2; IgE Total IgG	69% lower FECs; 26% lower burdens 56% lower FECs, 32% lower burdens	Bethony *et al.* (2005) Mendez *et al.* (2005)
r*Acey*-MTP-1/ r*Acey*-ASP-2	Quil-A (hamsters)	Total IgG	59% lower FECs; 36% lower burdens	Mendez *et al.* (2005)
r*Ac*-16	AS03 (beagles)	IgG1; IgG2; IgE; IFN-γ w/o IL-4 or IL-10 in PBCs	Significantly less blood loss; 63% lower FECs	Fujiwara *et al.* (2007)
r*Na*-ASP-2	Alhydrogel (humans)	Total IgG w/o IgE; proliferation of PBMCs	Generalized urticarial reactions in Phase I clinical trials	Bethony *et al.* (2008), Diemert *et al.* (2012)
r*Ac*-CP-2	AS02 (beagles) AS03 (beagles) ISA70 (beagles) Alum (beagles)	IgG1; IgG2; IgE IgG1; IgG2; IgE IgG1; IgG2; IgE IgG1; IgG2; IgE	~60% lower FECs ~60% lower FECs ~60% lower FECs ~60% lower FECs	Loukas *et al.* (2004)
r*Ac*-APR-1	AS03 (beagles) Alhydrogel (hamsters)	IgG1; IgG2; IgE; IFN-γ and proliferation of PBCs not evaluated	Significantly less blood loss; 70% lower FECs; 33% lower burdens 44% lower burdens	Loukas *et al.* (2005) Xiao *et al.* (2008)
r*Ac*-GST-1	AS03 (beagles) Alhydrogel (hamsters) Alhydrogel (hamsters)	IgG1; IgG2; IFN-γ and proliferation in PBCs not evaluated not evaluated	32% lower FECs; 39% lower burdens^b^ 54% lower burdens 51% lower burdens	Zhan *et al.* (2005) Zhan *et al.* (2005) Xiao *et al.* (2008)
*Acey-MEP-6*	pcDNA3·1+; Fugene 6 (hamsters)	Not evaluated	Marginally less blood loss and burdens	Wisniewski *et al.* (2013)
*Acey-MEP-7*	pcDNA3·1+; Fugene 6 (hamsters)	Total IgG	75% lower FECs; 50% lower burdens	Wisniewski *et al.* (2016)
*Ss-tmy-1*	Lambda Uni-zap XR vector; GM-CSF (mice)	Total IgG	None	Kerepesi *et al.* (2005)
*Ss-eat-6*	Lambda Uni-zap XR vector; GM-CSF (mice)	Total IgG	35% lower L3 survival within installed diffusion chambers	Kerepesi *et al.* (2005)
*Ss-lec-5*	Lambda Uni-zap XR vector; GM-CSF (mice)	None	None	Kerepesi *et al.* (2005)
*Ss-tmy-1*/*Ss-eat-6*/*Ss-lec-5*	Lambda Uni-zap XR vector; GM-CSF (mice)	Total IgG	None	Kerepesi *et al.* (2005)
r*Ss*-TMY-1	Alum (mice)	Total IgG	None	Abraham *et al.* (2011)
r*Ss*-EAT-6	Alum (mice)	Total IgG	None	Abraham *et al.* (2011)
r*Ss*-LEC-5	Alum (mice)	Total IgG	None	Abraham *et al.* (2011)
r*Ss*-NEI-1	Alum (mice)	Total IgG	None	Abraham *et al.* (2011)
r*Ss*-IR	Alum (mice)	Total IgG	80% lower L3 survival within installed diffusion chambers	Abraham *et al.* (2011)
r*Sr*-HSP60	CFA (rats) Alum (rats) none (rats)	IgM; IgG1; IgG2b; IgG2c; IFN-γ w/o IL-13 or proliferation in SCs IgM; IgG1; IgG2b; IgG2c; IL-13 and proliferation in SCs w/o IFN-γ IgM; IgG1; IgG2b; IgG2c; IFN-γ w/o IL-13 or proliferation in SCs	Slightly higher burdens 67% lower burdens Slightly higher burdens	Ben Nouir *et al.* (2012)
r*Hc*-H11	bac (sheep)	Antibody response	30% lower burdens	Reszka *et al.* (2007)
r*Hc*-MEP1	GST-fusion; Quil-A (sheep)	Antibody response	None	Smith *et al.* (2003)
r*Hc*-MEP3	GST-fusion; Quil-A (sheep)	Antibody response	None	Smith *et al.* (2003)
r*Hc*-hmcp-1/r*Hc*-hmcp-4/r*Hc*-hmcp-6	*E. coli* extract; GST-fusion; Quil-A (sheep)	Total IgG	38% lower burdens	Redmond and Knox, (2004)
r*Oo*-ASP-1	Quil-A (cattle)	Antibody response	None	Geldhof *et al.* (2008)
r*Oo*-PA	Quil-A (cattle)	Mucosal IgG1; mucosal IgG2	None	Vercauteren *et al.* (2004)
r*Tci*-CF-1/r*Tci*-MEP-1/r*Tci*-ES20/ r*Tci*-ASP-1/r*Tci*-SAA-1/r*Tci*-MIF-1/ r*Tci*-APY-1/r*Tci*-TGH	Quil-A (sheep)	IgG; IgA; mucosal IgG; mucosal IgA	70% lower FECs; 55% lower burdens	Nisbet *et al.* (2013)
r*As*14	CTB (mice)	IgG1; IgE; mucosal IgA	60% lower larval burdens in the lungs	Tsuji *et al.* (2001)
r*As*16	CTB (mice) CTB (pigs)	IgG1; IgG2a; IgG3; IgE; IFN-γ, IL-2 and IL-10 w/o IL-4 in SCs IgG1; IgE; mucosal IgA; IFN-γ, IL-4 and IL-10 in PBMCs	>60% lower larval burdens in the lungs >60% lower larval burdens in the lungs	Tsuji *et al.* (2003) Tsuji *et al.* (2004)
r*As*24	CFA (mice)	IgG1; IgG2a; IgG2b; IFN-γ and IL-10 w/o IL-4 in SCs	58% lower larval burdens in the lungs	Islam *et al.* (2005*a*)
r*As*37	CFA (mice)	Total IgG	none	Tsuji *et al.* (2002)
r*As*-PPase	TiterMax Gold (mice)	IgG1; IL-10 w/o IFN-γ, IL-2 or IL-4 in SCs	>70% lower larval burdens in the lungs	Islam *et al.* (2005*b*)
*AsEnol*	pVEX I vector (mice)	Total IgG; IFN-γ and proliferation in SCs	44% lower larval burdens in the lungs	Chen *et al.* (2012)
r*Bs*-Ag1	CFA (mice)	Total IgG	>60% lower larval burdens in the lungs	He *et al.* (2012)
r*Bs*-Ag2	CFA (mice)	Total IgG	>60% lower larval burdens in the lungs	He *et al.* (2009)
r*Bs*-Ag3	CFA (mice)	Total IgG	>60% lower larval burdens in the lungs	Wang *et al.* (2008)
r*Bs*-PYP-1	CFA (mice)	IgG1; IL-4 and IL-10 w/o IFN-γ or IL-2 in SCs	70% lower larval burdens in the lungs	Xie *et al.* (2013)
*Ts87*	*S. enterica* Typhimurium (mice)	IgG1; IgG2a; mucosal IgA; IFN-γ, IL-10, IL-5, IL-6, and IL-4 in SCs	~30% lower burdens	Yang *et al.* (2010*b*)
*TsGp43*	*S. enterica* Typhimurium (mice)	IgG1; IgG2a; IFN-γ and IL-5 in SCs and mLNs	~50% lower burdens	Pompa-Mera *et al.* (2011)
*TsPmy*	*S. enterica* Typhimurium (mice)	IgG1; IgG2a; mucosal IgA; IFN-γ, IL-2, IL-4, IL-5, IL-6 and IL-10 in SCs and mLNs	~45% lower burdens	Wang *et al.* (2016)
*Ts-NBLsp*	pcDNA3·1+	IgG1; IgG2a; elevated peripheral CD8^+^ T cells; total peripheral IFN-γ, IL-10 and IL-4	~78% lower larvae recovered in muscle tissue	Xu *et al.* (2017)

FECs, fecal egg counts; PBCs, peripheral blood cells; PBMCs, peripheral blood mononuclear cells; GM-CSF, granulocyte-macrophage colony-stimulating factor; SCs, spleen cells or spenocytes; bac, baculovirus-insect cell extract; PA, polyprotein allergen; CTB, cholera toxin B subunit; CFA, complete Freund's adjuvant; mLNs, mesenteric lymph nodes; ns, not significant; w/o, without.

a Results are compared with adjuvant alone control.

b These results were not statistically significant.

It is generally accepted that vaccines for STHs would be the most ideal control agents as they would prevent re-infection, which anthelmintic drugs do not. However, there are no licensed vaccines. Currently, chemotherapy is delivered *via* mass drug administration (MDA), stirring grave concern for the strong selection of drug resistance, especially with limited efficacious anthelmintic drugs (Geerts and Gryseels, 2001). Immunity to STH infections does not develop upon clearance, and represents a vast issue for humans who are rapidly re-infected after chemotherapy (Bethony *et al.* 2006). Thus, vaccines would go a long way towards STH elimination.

An important distinction between STH infections in humans and livestock, ruminants in particular, is that in the latter, protective immunity often develops with age, but in the former, this is not the case. So, although the duration of vaccine-induced protection in livestock may only need to last long enough to protect younger animals, for humans this protection would need to last throughout their lifetime. Thus, developing vaccines for humans will likely be a much greater challenge than for livestock due to the high likelihood that repeated immunizations will be needed throughout life.

What level of efficacy is needed to justify inclusion of an STH vaccine in a control program? Bartsch *et al.* (2016) developed a target product profile (TPP) for a recombinant subunit vaccine against human hookworms and evaluated the vaccines economic and epidemiologic impact on hookworm infection in endemic Brazil. A modelled human hookworm vaccine administered in a single dose to infants (78% coverage rate) with an efficacy of 80% at preventing L3 maturation and providing at least 10 years of protection with a single booster at 15 years of age (78% compliance), and costing $1 per dose, was highly cost-effective and economically dominant compared with no intervention or annual MDA (Bartsch *et al.* 2016). Thus, a human STH vaccine with such a TPP should be ideal.

For livestock, a subunit vaccine called Barbervax, developed in Australia, has been commercialized for *Haemonchus contortus* (barber's pole worm) that consists of a complex combination of native antigens from the worm's gut (described in more detail below). Barbervax is about 70% effective at reducing worm burdens and about 90% effective at reducing the number of eggs in the feces, which greatly reduces egg numbers throughout the pasture, and thus, the number of infections later on. Barbervax is much more cost-effective and efficacious compared with no intervention and MDA alone (especially considering rampant anthelmintic drug resistance in *H. contortus*) even though it has to be given repeatedly during the summer months (when infection is highest) because a protective memory response is not induced in vaccinated lambs during challenge infection. Clearly any STH vaccine for livestock with such a high efficacy would meet the requirements of an established TPP. As Barbervax is not a recombinant subunit vaccine, it requires numerous sheep to make enough worm guts to produce the vaccine. It is hard to tell if recombinant subunit vaccines for livestock should have similar TPPs and this will have to be addressed in the future.

There have been many attempts to develop vaccines for STHs of humans and livestock (Hewitson and Maizels, 2014). These parasites are relatively large, multicellular, cuticularized animals (i.e. unique from other infectious agents). They have relatively large genomes and produce many thousands of offspring per day, contributing to their rapid evolution (Zarowiecki and Berriman, 2015). It is thus clear that the biology of these parasites provides a formidable barrier for vaccine development. Also, although ‘holistic’ vaccines [radiation-attenuated larvae, crude worm extracts and excretory/secretory (ES) products] have provided high levels of protection against many STHs tested, with a few STHs of livestock being some exceptions (Hewitson and Maizels, 2014), because these parasites cannot be cultured on a large enough scale for mass vaccine administration (unlike bacteria and viruses) due to high costs, low stability and shelf-life, among other reasons, recombinant subunit vaccines are necessary. Hence, in this review we do not evaluate the previous vaccine studies that used ‘holistic’ vaccines as this has been extensively reviewed before (Hewitson and Maizels, 2014). Rather, we provide an update and evaluation of the previous studies that used recombinant subunit vaccines, the only realistic type of vaccine possible for controlling STHs.

Below, we first provide a general overview of the immune response(s) that are most relevant for this review. We then summarize and interpret the previous studies that used recombinant subunit vaccines for the major STHs of both humans and livestock (data presented in Table 2), grouped into separate sections based on their phylogenetic relationships (Blaxter and Koutsovoulos, 2015). In this review, we use the phylogeny of Nematoda that groups the phylum into five distinct clades (clades I, II, III, IV and V), of which the parasitic species relevant for human and veterinary medicine belong to clades I, III and V (Blaxter and Koutsovoulos, 2015). Included in each section is a brief description of each STH's basic biology to compare and contrast with the other STHs. We then provide an evaluation of all of these studies as a whole, and discuss potential avenues that may help to develop more efficacious, recombinant subunit vaccines for these critical parasites.

### T_H_1 and T_H_2 immune responses

Two key arms of the adaptive immune system are the type 1 and type 2 T helper (T_H_) cell responses (T_H_1 and T_H_2) (Romagnani, 2000). The T_H_1 response promotes cell-mediated immunity, while T_H_2 promotes humoral immunity. Whether a T_H_1 or T_H_2 response is induced generally depends on whether the pathogen is intracellular (such as viruses, and some bacteria and protozoa), or extracellular, such as helminths, and also toxins. However, it must be noted that many cracks have been discovered in the foundation for the T_H_1/T_H_2 paradigm, in particular that the abundance of effector cytokines, chemokines, co-stimulatory molecules, signalling pathways and transcription factors involved in each response is much greater and more complex than originally thought (Zhu *et al.* 2010), and there appear to be variations of these responses for particular pathogen types. But for the purpose of this review, we will only mention how T_H_1 and T_H_2 responses are mounted, briefly summarizing that covered in Kaiko *et al.* (2008).

We begin with the T_H_1 response. Host cells (non-professional antigen presenting cells or APCs) infected with intracellular pathogens present antigens *via* Class I Major Histocompatibility Complex (MHC) molecules. Cognate naïve CD8^+^ cytotoxic T (T_C_) cells then recognize the antigen-bound Class I MHC *via* the antigen-specific T cell receptor (TCR), resulting in the activation and differentiation of T_C_ cells into antigen-specific effectors that destroy the infected cell, or into memory T_C_ cells for responding to future cognate antigen. Professional APCs such as macrophages or dendritic cells also recognize and endocytose the pathogen or its antigens and present them on Class II MHC molecules. The antigen-loaded professional APCs then travel to nearby lymphoid tissues where they are recognized by cognate naïve CD4^+^ T_H_ (T_H_0) cells *via* antigen-specific TCR. In combination with the cytokine interleukin (IL)-12 secreted by the professional APCs and necessary co-stimulatory molecules (‘signal two’ and ‘signal three’), T_H_0 cells differentiate into T_H_1 effector cells that proliferate and produce massive quantities of pro-inflammatory cytokine interferon gamma (IFN-γ), as well as IL-2 and tumour necrosis factor (TNF), triggering the influx of inflammatory cells and the complement system into the site of infection, or into T_H_1 memory cells. T_H_1 effector cells also provide help to differentiating T_C_ cells and induce B cell-secretion of opsonizing antibodies or immunoglobulins (Ig), in general IgG2, which along with the complement system enhances phagocytosis, and thus, pathogen clearance (Kaiko *et al.* 2008).

When professional APCs present antigens from extracellular pathogens such as helminths, or toxins/allergens, to T_H_0 cells in lymphoid tissues, the professional APCs secrete IL-2 and IL-4 that, along with the necessary co-stimulators, drives differentiation of T_H_0 cells into T_H_2 effectors or into T_H_2 memory cells. T_H_2 effector cells secrete three key cytokines that drive classic features of the T_H_2 response, IL-4, IL-5 and IL-13. They can also secrete IL-10, which tends to dampen immune responses. IL-4: (1) drives antibody class switching from IgM to IgG1 and IgE; (2) drives affinity maturation by somatic hypermutation in B cells; (3) stimulates B cell proliferation and differentiation into plasma cells; and (4) drives differentiation of T_H_2 cells, thus providing feedback regulation. IL-5 attracts and activates eosinophils, and also drives the secretion of IgA (Lebman and Coffman, 1988), the most abundant mucosal antibody responsible for intestinal homoeostasis (Mantis *et al.* 2011). IL-13 mediates the release of granules from basophils and mast cells and can also drive class switching to IgE. In T_H_2-mediated allergy, IgE is key, with the Fcε region of the heavy chain recognized with high affinity by the FcεRI receptor that is constitutively expressed on basophils and mast cells. This receptor is expressed at much lower levels on professional APCs and is inducible on eosinophils. Basophils, mast cells and eosinophils are granulocytes that collectively release proinflammatory mediators and cytotoxins that result in a robust, hypersensitivity reaction associated with allergy (Kaiko *et al.* 2008; Stone *et al.* 2010).

T_H_2-type responses are clearly associated with reduced intensities of infection by STHs (Anthony *et al.* 2007; Nair and De'Broski, 2016). These responses involve an interconnected, complex network of IgE-driven mastocytosis and basophilia, IL-5-driven eosinophilia, and IL-4/IL-13-driven alternative activation and recruitment of macrophages and parasite expulsion. Expulsion occurs *via* intestinal epithielial cell (IEC) secretion of RELM-β and Muc2/Muc5ac (‘mucus’), IEC turnover (‘epithelial escalation’) and/or increased IEC permeability and smooth muscle contraction (‘weep and sweep’ response) (Anthony *et al.* 2007; Nair and De'Broski, 2016). However, subunit vaccines do not necessarily need to induce the natural immune response(s) in order to provide protection. Subunit vaccines for STHs have focused primarily on the idea that the protein antigens targeted by the vaccines are important for infection, rather than simply being immunogenic in order to direct the immune system to the pathogen to attack it. Immunization with most STH antigens triggers IgG responses (IgG1 and IgG2 are by far the most abundant serum antibodies), although titre is highly variable. The common theme is that high IgG titre blocks antigen function, and if the antigen is essential, protection may be achieved. As discussed below, the most promising vaccine candidates discovered from previous studies are consistent with this common theme.

## RECOMBINANT SUBUNIT VACCINES FOR STRONGYLIDS

The order Strongylida (Nematoda clade V) contains many important parasitic species, including devastating parasites of humans, livestock, dogs and cats, as well as species that infect birds, reptiles and amphibians (Durettedesset *et al.* 1994). Strongylida was once grouped into four suborders: Strongylina, Ancylostomatina, Trichostrongylina and Metastrongylina, of which the former three suborders contained the most significant species for human and veterinary medicine (Durettedesset *et al.* 1994). But it is now clear that these suborders are not monophyletic and that the most significant species within the former three suborders are actually in their own monophyletic group (Chilton *et al.* 2006). Thus, we do not use the suborders in this review, but only refer to the most relevant families, which are currently stable. The most extensive studies with recombinant subunit vaccines for strongylids have been on hookworms, with studies on *Strongyloides* spp. and a few strongylid parasites of livestock also prevalent in the literature. As many of the important strongylid species within these families have relatively recent common ancestries with very similar modes of infection (Durettedesset *et al.* 1994; Chilton *et al.* 2006; Blaxter and Koutsovoulos, 2015), a single, pan-strongylid vaccine with efficacy against a majority of the species seems within reason, hence why we have grouped them into their own section.

### Hookworms

Hookworms (family Ancylostomatidae) are a serious issue for human health, as well as for dogs and cats, and of all the STHs, these parasites cause the most severe disease due to their voracious blood feeding (Table 1). The human hookworms (*Necator americanus*, *Ancylostoma duodenale* and *Ancylostoma ceylanicum*) infect almost 500 million people (Pullan *et al.* 2014). *Necator americanus* and *A. duodenale* infect predominantly humans while *A. ceylanicum* infects humans, dogs, cats and some rodents. Third stage larvae (L3) penetrate the skin and enter the blood stream, arriving in the lungs for L3 maturation (*N. americanus* and *Ancylostoma* spp.). L3 then migrate up the trachea into the pharynx and are swallowed. *Ancylostoma* spp., but not *Necator*, are also able to directly enter orally. After passing through the stomach, L3 burrow into the epithelium of the small intestine, most often in the duodenum, and rupture capillaries to feed on blood. Blood feeding by the attached hookworms causes iron deficiency anaemia in humans, and is of major concern for children and pregnant women. Adult hookworms mate within the lumen of the small intestine, and each female can produce over 10 000 eggs per day. These hematophagous parasites can live for years in the same host (Hotez *et al.* 2004).

Pre-clinical vaccine studies for hookworms have either used the dog hookworm *Ancylostoma caninum*-beagle or *A. ceylanicum-*golden hamster (*Mesocricetus auratus*) pathosystems, or a laboratory strain of *N. americanus* adapted to golden hamsters. Antigens from both infectious L3 and adult hookworms have been subjected to pre-clinical vaccine studies. These past attempts focused on recombinant subunit vaccines using *Escherichia coli*, *Pichia pastoris* or baculovirus-insect cell expression systems.

#### Secreted L3 antigens

Beagles that received four intramuscular (i.m.) injections at 140 *µ*g each of a recombinant, *E. coli*-expressed Astacin-like metalloprotease (r*Ac*-MTP-1), natively secreted by L3, and formulated with GlaxoSmithKline's AS02 adjuvant (T_H_1-biased) in general had lower *A. caninum* worm burdens and fecal egg counts (FECs) compared with AS02 alone [Table 2; the level of protection was correlated with serum IgG2 endpoint titre (i.e. the inverse of the greatest serum dilution at which there is an above background response)] (Hotez *et al.* 2003). This initial study provided proof of concept that recombinant subunit vaccines have efficacy against hookworms.

*Ancylostoma* secreted protein (*Ac*-ASP-2) is natively expressed in the oesophageal gland cells, basal lamina and cuticle, and secreted by L3. Baculovirus-insect cell-expressed r*Ac*-ASP-2 formulated with GlaxoSmithKline's AS03 adjuvant (unresolved T_H_ response) and injected i.m. four times into beagles at 100 *µ*g doses had average serum IgG1 and IgG2 endpoint titre of >10 000 and average serum IgE endpoint titre of 1000 (Table 2). r*Ac*-ASP-2/AS03 immunization resulted in an average 26 and 69% lower *A. caninum* worm burdens and FECs, respectively, compared with AS03 alone (Table 2), although the latter measure had extensive variation. Interestingly, in the same study, intensity of *N. americanus* infection in individuals from endemic Brazil was negatively correlated with IgE titre specific for *Na*-ASP-2, revealing this L3 antigen as naturally immunogenic and, possibly, naturally protective.

In another study, *P. pastoris*-expressed r*Acey*-MTP-1 and r*Acey*-ASP-2 were injected three times i.m. into golden hamsters at 25 *µ*g doses as both monovalent and bivalent vaccines formulated with Quil-A adjuvant (mixed T_H_1/T_H_2 response). Immunization resulted in impressive average total serum IgG endpoint titre (no IgG subclass-specific secondary antibodies for hamsters) between 50 000 and 200 000, including from both the monovalent and bivalent vaccines (Table 2), suggesting that immunological interference was not a problem. Similar to that found in the *A. caninum*-beagle pathosystem, both monovalent vaccines resulted in lower *A. ceylanicum* FECs (r*Acey*-ASP-2/Quil-A = 56% lower; r*Acey*-MTP-1/Quil-A = 43% lower) and worm burdens (r*Acey*-ASP-2/Quil-A = 32% lower; r*Acey*-MTP-1/Quil-A = 28% lower) compared with Quil-A alone, although the worm burden for r*Acey*-MTP-1/Quil-A were not significantly less (Table 2). Surprisingly, the bivalent vaccine did not provide significant, additive protection over the monovalents with an average 59 and 36% lower FECs and worm burdens, respectively, compared with Quil-A alone (Table 2). On the other hand, clinicopathological parameters (reduced haemoglobin and body weight) were significantly improved in the bivalent compared with the monovalent vaccines (Table 2) (Mendez *et al.* 2005). Nonetheless, lack of a significant additive effect on FECs and worm burdens suggests that ASP-2 and MTP-1 may have functional redundancies during tissue migration.

*Ac*-16 is homologous to larval surface antigens of Nematoda clade III helminths that had been previously shown to protect against experimental infections (see section on ascarids below). *Ac*-16 was expressed in *E. coli*, formulated with AS03 and injected three times i.m. into beagles at 100 *µ*g doses. Immunization of beagles with r*Ac*-16/AS03 resulted in peak serum IgG1, IgG2 and IgE endpoint titre of around 5000, 100 000 and 1000, respectively, significant r*Ac*-16-stimulated proliferation of peripheral blood cells and production of IFN-γ, but not IL-4 or IL-10 (Table 2). r*Ac*-16/AS03 also resulted in an average 63% lower *A. caninum* FECs and significantly less blood loss compared with AS03 alone, but worm burdens were similar (Table 2) (Fujiwara *et al.* 2007). Nonetheless, this study clearly demonstrated the vaccine efficacy of r*Ac*-16/AS03 for reducing hookworm fecundity and hookworm-associated blood loss, in association with IgG and IgE responses, and a T_H_1-biased cytokine profile (but without measuring IL-5 and IL-13, contribution from at least some T_H_2 cytokines cannot be ruled out). *Ac*-16 was also localized to the L3 cuticle with r*Ac*-16/AS03 serum from beagles (Fujiwara *et al.* 2007).

With the promising pre-clinical results for ASP-2 (significantly reduced worm burdens, FECs and clinical pathology), and negative correlation between infection intensity and *Na*-ASP-2-specific serum IgE responses in individuals from endemic Brazil, *P. pastoris*-expressed r*Na*-ASP-2 formulated on Alhydrogel (aluminum hydroxide gel; a widely used T_H_2 adjuvant) (Goud *et al.* 2005) advanced to clinical trials. Three i.m. injections in healthy and unexposed adults of either 10, 50 or 100 *µ*g doses were well tolerated and induced total serum IgG endpoint titre of around 10 000, while serum IgE endpoint titre were below 100, and significant r*Na*-ASP-2-stimulated proliferative responses in peripheral blood mononuclear cells (PBMCs) (Table 2) (Bethony *et al.* 2008). However, r*Na*-ASP-2/Alhydrogel in only a single injection of 10 *µ*g failed in Phase I as individuals previously infected with or exposed to *N. americanus* resulted in generalized urticarial reactions due to pre-existing *Na*-ASP-2-specific serum IgE (Table 2) (Diemert *et al.* 2012). Although r*Na*-ASP-2 is still being optimized to reduce its allergic potential, namely by removing the IgE epitopes and fusion with Fcγ1, thus promoting its ligation with the FcγRIIb inhibitory receptor (Zhan *et al.* 2012), these findings have halted vaccine studies focused on L3 antigens.

#### Adult intestinal antigens

Results with ASP-2 demonstrated an important problem for targeting antigens that are naturally immunogenic during hookworm infection (at least from L3); there may be an allergic reaction following immunization of individuals that had been previously infected or exposed. Thus, next attempts focused on the so-called ‘hidden antigens’ (i.e. STH proteins that are not exposed to the host immune system during infection but that may be accessed by host immune effectors induced by vaccination) present in the hookworm intestinal lumen and thus accessible to host antibodies in the ingested blood (Hotez *et al.* 2010). Hookworms have a semi-ordered proteolytic cascade in their intestinal lumen for digesting haemoglobin during blood meals (Williamson *et al.* 2004; Ranjit *et al.* 2009) and all of the vaccine studies pertaining to adult intestinal antigens have focused on this process (Hotez *et al.* 2010).

A recombinant, *P. pastoris*-expressed, Cathepsin B cysteine protease (r*Ac*-CP-2) that natively localizes in the hookworm intestinal lumen (Loukas *et al.* 2004) and that degrades fragments of haemoglobin *in vitro* (Williamson *et al.* 2004; Ranjit *et al.* 2009), was formulated with multiple adjuvants [AS02, AS03, ISA70 and aluminium phosphate (alum)] and injected i.m. three times into beagles at 100 *µ*g doses (Loukas *et al.* 2004). Although r*Ac*-CP-2/adjuvant immunizations resulted in average serum IgG1 and IgG2 endpoint titre of >10 000 and average serum IgE endpoint titre of around 1500 regardless of adjuvant, *A. caninum* worm burdens were the same as adjuvant controls, while FECs were an average 60% lower (Table 2). The lowest FECs and highest IgG titre were observed with the AS03 adjuvant, although the differences from the other adjuvants were not significant. Also, pooled IgGs from r*Ac*-CP-2/AS03-immunized beagles reduced the *in vitro* r*Ac*-CP-2 protease activity by an average of 73% and recognized native *Ac*-CP-2 within the adult hookworm intestinal lumen (Loukas *et al.* 2004), suggesting that blocking/neutralizing IgG contributed to the reduced fecundity.

Another adult antigen, Cathepsin D aspartic protease (*Ac*-APR-1), which is also localized within the intestinal lumen but degrades whole haemoglobin (Williamson *et al.* 2004; Ranjit *et al.* 2009), was recombinantly expressed in *P. pastoris*. r*Ac*-APR-1 was formulated with AS03 and injected i.m. three times in 100 *µ*g doses into beagles. r*Ac*-APR-1/AS03 immunization resulted in average serum IgG1 and IgG2 endpoint titre of >10 000 and average serum IgE endpoint titre of around 1500 (Table 2). There was significant IFN-γ production and proliferative responses in r*Ac*-APR-1-stimulated whole blood cells (Table 2). r*Ac*-APR-1/AS03 immunization resulted in 70 and 33% lower median *A. caninum* FECs and worm burdens, respectively, and anaemia was significantly less compared with AS03 alone (Table 2). Similar to r*Ac*-CP-2/AS03, serum IgG from beagles immunized with r*Ac*-APR-1/AS03 reduced the *in vitro* protease activity of r*Ac*-APR-1 by an average of 71%, and recognized native *Ac*-APR-1 within the adult hookworm intestinal lumen (Loukas *et al.* 2005).

The final protective adult hookworm antigen is a glutathione *S*-transferase (GST-1) that is localized within the intestine, muscular tissue and hypodermis and is also present in adult excretory/secretory (ES) products. GST-1 is believed to scavenge and detoxify haeme liberated from degraded haemoglobin within the hookworm intestine (Zhan *et al.* 2005). Based on GST-1's localization in the hypodermis and presence in ES products, the protein may have other functions as well. *Pichia pastoris*-expressed r*Ac*-GST-1/AS03 i.m. injections into beagles (three times in 100 *µ*g doses), as with r*Ac*-CP-2/AS03 and r*Ac*-APR-1/AS03, resulted in average serum IgG1 and IgG2 titre of >10 000, lower average serum IgE endpoint titre of <500, and significant proliferative responses and IFN-γ production in r*Ac*-GST-1-stimulated whole blood cells (Table 2). r*Ac*-GST-1/AS03 immunization resulted in an average 32 and 39% lower *A. caninum* FECs and worm burdens, respectively, compared with AS03 alone (Table 2). These reductions were not significant, which was attributed to too much variation between beagles. However, in the same study, r*Ac*-GST-1 formulated on Alhydrogel was subcutaneously (s.c.) injected three times at 25 *µ*g doses into golden hamsters and challenged with the adapted laboratory *N. americanus* strain, and worm burdens were a significant 54% lower than Alhydrogel alone (Table 2) (Zhan *et al.* 2005). Although immune correlates of this protection were not evaluated, this result provided proof of concept for cross-protection against hookworms from different genera using the same immunogen, in addition to revealing GST-1 as a promising vaccine target.

In another study using the *N. americanus*-golden hamster pathosystem, r*Ac*-GST-1/Alhydrogel under the same immunization regimen as Zhan *et al.* (2005) again had an average of 51% lower *N. americanus* worm burdens, and in the same study, r*Ac*-APR-1/Alhydrogel had 44% lower *N. americanus* worm burdens compared with Alhydrogel alone (Table 2) (Xiao *et al.* 2008). Thus, both *A. caninum* immunogens were capable of cross protecting against *N. americanus* in golden hamsters, although immune correlates of protection were not evaluated.

The cross-protections against *N. americanus* in golden hamsters with r*Ac*-GST-1/Alhydrogel and r*Ac*-APR-1/Alhydrogel are the greatest reported to date compared with any other recombinant subunit vaccines against hookworms. A recombinant bivalent subunit vaccine containing r*Na*-GST-1 expressed in *P. pastoris* and a catalytically inactive, but still immunogenic (Pearson *et al.* 2009), r*Na*-APR-1 expressed in tobacco plants (used as an alternative to *P. pastoris* since expression was low) and formulated on Alhydrogel is currently in Phase I clinical trials (Hotez *et al.* 2016). Clinical testing is also evaluating if including synthetic Toll-like receptor agonists glucopyranosyl lipid A (GLA) or CpG oligodeoxynucleotide will help achieve acceptable immunogenicity (Hotez *et al.* 2016). Surprisingly, to the best of our knowledge there are no published results on the level of protection provided by the bivalent vaccine. Some important questions remaining to be addressed are whether there is an additive or maybe synergistic effect when combining rAPR-1 and rGST-1, or rather, no additive effect, similar to that observed for the r*Acey*-ASP-2/r*Acey*-MTP-1 bivalent (Mendez *et al.* 2005). Given that both APR-1 and GST-1 are assumed to function in haemoglobinolysis (Hotez *et al.* 2010), it seems reasonable to suspect redundancy in blocking/neutralizing both antigens. For example, blocking/neutralizing APR-1 would in theory prevent liberation of heme since haemoglobin would no longer be broken down, thereby making GST-1 redundant. Moreover, adult hookworms can survive *in vitro* in serum alone, so it is likely that haemoglobin is not the only source of protein in their diet within the intestine. Nevertheless, this vaccine is admittedly not expected to prevent hookworms from establishing infection within the small intestine (Hotez *et al.* 2010; Hotez *et al.* 2016), but the evidence does suggest that it will reduce clinicopathological parameters below threshold levels. Thus, although there are still many unanswered questions for this vaccine, there is cautious optimism for significant impact.

#### DNA vaccines

More recently, a pair of studies used a DNA vaccine approach against *A. ceylanicum* in golden hamsters (Wisniewski *et al.* 2013, 2016). With a DNA vaccine, the antigen's cDNA is cloned into a plasmid vector and then the plasmid is injected into the host with the expectation that it will enter host cell nuclei for transcription and will subsequently be translated in the host cell cytoplasm. In the first study, *A. ceylanicum metalloprotease 6* (*Acey-MEP-6*) cDNA (a likely adult intestinal antigen) was cloned into the plasmid vector pcDNA3·1+, mixed with Fugene 6 Transfection Reagent (presumably to enhance uptake into host cells), and administered intranasally (i.n.) both once and three times in 50 *µ*g doses into golden hamsters. Single dose pcDNA3·1+/*Acey-MEP-6*/Fugene 6, but not triple dose, resulted in significantly lower *A. ceylanicum* worm burdens and significantly higher haematocrit compared with golden hamsters receiving no vaccine, but not compared with golden hamsters receiving either single or triple doses of pcDNA3·1+/Fugene 6 (empty vector/transfection reagent) control (Table 2) and immune responses were not evaluated (Wisniewski *et al.* 2013). Thus, although pcDNA3·1+/*Acey-MEP-6*/Fugene 6 appeared to have some vaccine efficacy, the clear impact that the empty vector/transfection reagent control had on *A. ceylanicum* challenge infection and lack of evaluated immune correlates of protection makes it difficult to interpret these results.

In a follow up study with an *Acey-MEP-7* cDNA (paralog of *Acey-MEP-6*), single dose pcDNA3·1+/*Acey-MEP-7*/Fugene 6 (amount of plasmid was not reported) resulted in significantly lower *A. ceylanicum* worm burdens and FECs, and significantly higher post-challenge hematocrit compared with pcDNA3·1+/Fugene 6 control in golden hamsters (Table 2) (Wisniewski *et al.* 2016). Also, pcDNA3·1+/*Acey-MEP-7*/Fugene 6 resulted in significantly greater r*Acey-*MEP-7-specific total serum IgG responses (measured as optical density at 450 nm; OD450) compared with pcDNA3·1+/Fugene 6 control, although the difference was not great (average OD450 of ~0·7 compared with ~0·4) (Table 2) (Wisniewski *et al.* 2016). Although this study clearly indicates significant vaccine efficacy of pcDNA3·1+/*Acey-MEP-7*/Fugene 6, the issue with the empty vector/transfection reagent control also having an effect (Wisniewski *et al.* 2013) has not been fully resolved, and immune correlates are weak. Thus, efficacy of DNA vaccines for hookworms remains unclear.

### Strongyloides *spp.*

Species within the *Strongyloides* genus (family Strongyloididae) have been reported to infect humans, non-human primates, dogs, cats and some rodents (Table 1). At least two species, *Strongloides stercoralis* and *Strongyloides fuelleborni*, commonly infect humans, with a total of approximately 100 million people infected (Puthiyakunnon *et al.* 2014). Although symptoms are often subclinical, strongyloidiasis can be deadly in immunosuppressed people with hyperinfection syndrome. These strongyloid parasites alternate between free-living and parasitic cycles. Free-living larvae passed in the stool can either moult to infectious L3 or can continue a free-living cycle and ultimately moult to egg-laying adult worms outside of a host for a single generation (an advantage in the absence of a suitable host). Similar to hookworms, in the presence of a suitable host, L3 penetrate the skin and enter the bloodstream where they undergo maturation in the lungs, followed by migration up the trachea and into the pharynx where they are swallowed, finally developing into adult worms within tunnels of the small intestine. Eggs hatch within the small intestine, and larvae are either excreted with the feces outside of the host, or can develop to infectious L3 within the small intestine and immediately reinfect the intestinal mucosa (autoinfection). Chronic asymptomatic infections can be maintained in human hosts for decades (Puthiyakunnon *et al.* 2014).

#### DNA vaccines

The first study that tested recombinant subunit vaccines against *Strongyloides* spp. used a DNA vaccine approach against experimental *S. stercoralis* L3 infection in BALB/cJ mice (Kerepesi *et al.* 2005). *Strongloides stercoralis* deoxycholate-soluble L3 antigens tropomyosin (*Ss*-TMY-1), Na+-K+ ATPase (*Ss*-EAT-6) and galectin (*Ss*-LEC-5) were shown to be recognized by serum IgG isolated from human plasma obtained from exposed Haitian donors, and this serum was shown to passively transfer protection to BALB/cJ mice (Kerepesi *et al.* 2004). Six intradermal (i.d.) injections of 20 *µ*g doses were performed in the ears of mice with Lambda Uni-zap XR vector individually containing cDNA inserts for all three *S. stercoralis* antigens mixed with the plasmid VC1701 containing murine ganulocyte-macrophage colony-stimulating factor (GM-CSF). Only plasmid *Ss-tmy-1/GM-CSF* and plasmid *Ss-eat-6/GM-CSF* resulted in significant, synthetic peptide-specific total serum IgG responses compared with control plasmid *GM-CSF* alone, albeit with very weak responses (OD450 of ~0·25 and ~0·6, respectively, compared to ~0·15 in control using 1 : 100 serum dilutions) (Table 2). Immunized mice were challenged s.c. with *S. stercoralis* L3 in diffusion chambers (i.e. devices with openings that are small enough to keep the worms inside but large enough to allow immune cells to enter) for 4 days, and only plasmid *Ss-eat-6/GM-CSF* resulted in significantly lower L3 survival (35%) within the diffusion chambers compared with plasmid *GM-CSF* control (Table 2). However, mice immunized with a combination of all three DNA vaccines did not result in significantly lower L3 survival compared with plasmid *GM-CSF* control (Table 2). Although triply vaccinated mice had significant total serum IgG responses against Ss-EAT-6 synthetic peptides, these responses were lower than plasmid *Ss-eat-6/GM-CSF* alone. Passive transfer of serum from vaccinated into naïve mice for all DNA vaccine groups did not result in significantly lower *S. stercoralis* L3 survival compared with plasmid *GM-CSF* control serum, not even for plasmid *Ss-eat-6/GM-CSF*, although the latter did result in the lowest survival (Kerepesi *et al.* 2005).

#### Protein vaccines

*Ss*-TMY-1, *Ss*-EAT-6 and *Ss*-LEC-5, as well as two highly and naturally immunogenic *S. stercoralis* antigens used for diagnostics in humans [*Ss*-NEI-1 and *Ss*-IR; (Ramanathan *et al.* 2008)] were tested for expression in *E. coli*, baculovirus-insect cells, and the yeast *Kluyveromyces lactis* expression systems (Abraham *et al.* 2011). All but r*Ss*-TMY-1 expressed well in the baculovirus-insect cell expression system, while r*Ss*-TMY-1 expressed well in *K. lactis*. All immunogens were formulated with alum and injected s.c. twice in 25 *µ*g doses into BALB/cByJ mice. All vaccines resulted in total serum IgG responses against cognate immunogen (albeit weak; ODs between only 0·5 and 2·0 at 1:100 serum dilutions, with background in controls around 0·25) (Table 2). Cellular immune responses were not significantly different for any of the vaccines, measured as the number of neutrophils, macrophages and eosinophils infiltrated into diffusion chambers after challenge. Of all the vaccines tested, only immunization with r*Ss*-IR/alum resulted in significantly lower survival of *S. stercoralis* L3 with an average 80% lower survival in three different trials compared with alum alone (Table 2). Passive transfer of serum from mice immunized with r*Ss*-IR/alum into naïve mice again resulted in an approximately 80% lower L3 survival. Finally, serum from mice immunized with r*Ss*-IR/alum localized native *Ss*-IR within granules of the glandular oesophagus in immunoelectron microscopy, and on the surface of *S. stercoralis* L3 in immunoconfocal microscopy (Abraham *et al.* 2011).

Consistent with DNA vaccination (Kerepesi *et al.* 2005), neither *Ss*-TMY-1 nor *Ss*-LEC-5 showed any evidence of efficacy as recombinant, protein-based, subunit vaccines (Abraham *et al.* 2011). Although the lack of significant vaccine efficacy for *Ss*-EAT-6 (Abraham *et al.* 2011) somewhat contradicts its efficacy (albeit low) as a DNA vaccine (Kerepesi *et al.* 2005), it was speculated that r*Ss*-EAT-6 might need a different adjuvant to induce protection. On the other hand, r*Ss*-IR/alum is a potential candidate whose efficacy as a recombinant subunit vaccine for strongyloidiasis (Abraham *et al.* 2011) in principle could be further investigated. However, the weak IgG response, the lack of significant immune correlates with r*Ss*-IR/alum-induced protection, and the presence of *Ss*-IR on the surface of L3 (potentially risking allergic reactions in clinical trials as was found with r*Na*-ASP-2 detailed above) present important hurdles for this vaccine. Abraham *et al.* (2011) speculate that the mechanism of r*Ss*-IR/alum-induced protection may be through complement and/or antibody-dependent cellular cytotoxicity (ADCC) as these mechanisms were demonstrated to be important for vaccine-induced reduction of *S. stercoralis* L3 survival (Ligas *et al.* 2003; Kerepesi *et al.* 2004). Although these mechanisms may be important for r*Ss*-IR/alum-induced protection in the *S. stercoralis*-mouse experimental model with implanted diffusion chambers, it seems plausible that such mechanisms may not be sufficient to prevent the establishment of patent infections within the small intestine, and more thorough studies are needed.

Heat shock protein 60 (HSP60) from the rodent parasite *Strongyloides ratti* is a naturally immunogenic antigen during infection in C57BL/6 mice that has been shown to be recognized by IgM and to stimulate proliferative responses in spleen and mesenteric lymph node (mLN) cells (Ben Nouir *et al.* 2012). r*Sr*-HSP60 expressed in *E. coli* was emulsified with complete Freund's adjuvant (CFA; T_H_1-biased), formulated with alum, or used without adjuvant, and injected i.p. twice at 100 *µ*g doses. Immunization with r*Sr*-HSP60/CFA, and interestingly, r*Sr*-HSP60 alone induced a skewed T_H_1 response characterized by significant IgM, IgG1, IgG2b/c, and IFN-γ, without IL-13 or significant proliferative splenocyte responses (Table 2). r*Sr*-HSP60 alone was also found to increase IFN-γ production in DO11·10 mouse splenocytes *in vitro* stimulated with cognate ovalbumin peptide. Not only did either vaccine fail to protect against challenge infection, but also immunization with r*Sr*-HSP60/CFA or r*Sr*-HSP60 alone actually slightly increased susceptibility. Conversely, although immunization with r*Sr*-HSP60/alum resulted in similar IgM, IgG1 and IgG2b/c responses compared with r*Sr*-HSP60/CFA and r*Sr*-HSP60 alone, significant proliferation and production of IL-13 without IFN-γ in splenocytes was observed after r*Sr*-HSP60 stimulation (Table 2). Immunization with r*Sr*-HSP60/alum resulted in >50% protection against *S. ratti* challenge infection (Table 2) (Ben Nouir *et al.* 2012). IgE responses were not evaluated, but given the association between IL-13 and protection, it seems plausible that IgE was involved. These findings clearly indicate that a T_H_2 adjuvant such as alum can overrule an antigen's intrinsic induction of T_H_1 and provide significant protection against a STH infection.

### Trichostrongyloids (family Trichostrongylidae) of livestock

#### Haemonchus contortus

*Haemonchus contortus* is a worldwide trichostrongyloid of small ruminants, mostly sheep and goats, with a life cycle similar to hookworms except that L3 attach to the abomasal mucosa in the ruminant stomach (abomasum) to feed on blood (Zajac, 2006) (Table 1). Targets of subunit vaccines for *H. contortus* have focused primarily on three different integral membrane protease complexes located within the worm's intestine, H11 aminopeptidase, galactose-containing glycoprotein (H-gal-GP) and Thiol Sepharose-Binding Protein (TSBP). In all cases, vaccine-induced immunity is believed to be due to blocking/neutralizing antibodies. Although high levels of protection have not yet been achieved with any recombinant subunit vaccine against haemonchosis, various technical factors are likely accountable and further studies are needed.

An adult *H. contortus* protein extract enriched and sub-fractionated for the H11 aminopeptidase complex resulted in an average 71% lower *H. contortus* FECs and worm burdens in Merino sheep compared with adjuvant control (Munn *et al.* 1993). An H11 aminopeptidase component was cloned and expressed in the baculovirus-insect cell expression system (Reszka *et al.* 2007). Recombinant H11 aminopeptidase-expressing baculovirus-infected insect cell extracts (bac-r*Hc*-H11) – used instead of purified protein due to anticipated immunostimulatory effects of insect cell extracts – were injected twice i.m. in estimated 300 *µ*g doses into Merino sheep. Serum antibody responses (no mention of classes) were monitored throughout infection and responses were higher than bacWT for bac-r*Hc*-H11 after immunization, although the difference was not great (OD of ~2·25 for bac-r*Hc*-H11 with a background OD of ~1·0 for bacWT; responses were low despite lack of serum dilution). Sheep immunized with bac-r*Hc*-H11 had a significant 30% lower *H. contortus* worm burdens compared with bacWT control (Table 2) (Reszka *et al.* 2007). Although this study indicated significant vaccine efficacy of bac-r*Hc*-H11 against *H. contortus* challenge in sheep, adjuvanted, purified recombinant protein, as well as other aminopeptidase components of the H11 complex, may provide greater protection, and further investigation is warranted.

H-gal-GP is a detergent-soluble, heavily aspartyl and metalloproteolytic complex containing 12 different proteins, mostly pepsins, metalloproteases and cysteine protease-like enzymes, as well as a cysteine protease inhibitor and thrombosponin, that when adjuvanted with CFA provided immunized Suffolk–Greyface cross lambs 72% lower *H. contortus* worm burdens and 93% lower FECs compared with CFA alone (Smith *et al.* 1994; Longbottom *et al.* 1997; Redmond *et al.* 1997; Smith *et al.* 1999; Skuce *et al.* 2001). SDS–PAGE separation of H-gal-GP determined that the isolated, individual components adjuvanted with CFA are much less protective alone (Smith and Smith, 1996). Moreover, reduced and urea-denatured H-gal-GP and the most protective metalloprotease components, as well as their recombinant forms in *E. coli*, all adjuvanted with Quil-A, eliminated significant protection against *H. contortus*, while native H-gal-GP/Quil-A still resulted in protection similar to the previous studies (Table 2) (Smith *et al.* 2003). These findings demonstrate that vaccine-induced protection with H-gal-GP, whether adjuvanted with CFA or Quil-A, most likely requires a complex combination of conformational epitopes provided only by the complex, which has been a major challenge for its application as a recombinant subunit vaccine for haemonchosis. The use of random peptide phage-display libraries has been suggested as a potential method to identify peptide sequences in H-gal-GP that potentially mimic the antigenic epitopes (Ellis *et al.* 2012).

TSBP is another intestinal, membrane-bound glycoprotein complex exposed to the luminal surface, and is enriched with cysteine proteases (Knox *et al.* 1999), drawing parallels with the partially protective *A. caninum* intestinal cysteine protease reported by Loukas *et al.* (2004). Suffolk–Greyface cross-lambs injected three times i.m. with TSBP formulated with CFA, IFA, or Quil-A at 200 *µ*g doses had 95, 54 and 82% lower *H. contortus* FECs, and 52, 43 and 45% lower adult worm burdens, respectively, compared with adjuvant controls (reduction in adult worm burdens for TSBP/IFA was not significant) (Knox *et al.* 1999). The TSBP intestinal membrane complex was therefore determined as a highly protective immunogen when formulated with either CFA or Quil-A, but surprisingly, protection was lesser with IFA, a classic T_H_2 adjuvant.

Immunoscreening of an *H. contortus* adult, gut-derived cDNA library with TSBP antisera recognized three Cathepsin-B-like cysteine proteases (hmcp-1, 4 and 6), and hmcp-1, 4 and 6 were immunolocalized to the gut microvillar surface and found to be exclusively expressed during blood-feeding (Skuce *et al.* 1999). Consistently, TSBP further fractionated for cysteine proteases, formulated with Quil-A, and injected i.m. resulted in similar levels of protection compared with the unfractionated TSBP complex with ~30% lower *H. contortus* worm burdens in trial 1 and ~46% lower in trial 2, even with only 3 *µ*g doses per injection of the former and 100 *µ*g for the latter, and the non-cysteine protease TSBP fraction failed to protect (Redmond and Knox, 2004). Also, although the TSBP cysteine protease fraction resulted in slightly higher numbers of abomasal eosinophils and mast cells, as well as serum IgE and IgA, but not serum IgG endpoint titre, compared with unfractionated TSBP and the non-cysteine protease TSBP fraction, only serum IgG titre slightly correlated with protection between individual lambs within the group. For the TSBP cysteine protease fraction, not only did total serum IgG titre slightly negatively correlate with FECs, but also an equal balance between IgG1 and IgG2 subclasses appeared to be most protective, though this conclusion did not hold up in a second trial. Importantly, although recombinant, *E. coli*-expressed, trivalent hmcp-1, 4 and 6 expressed as GST-fusions in *E. coli* cell extracts (~80–90% pure proteins), formulated with Quil-A, and injected three times i.m. at 100 *µ*g doses failed to lower *H. contortus* FECs in immunized lambs compared with Quil-A alone, adult worm burdens were 38% lower and along with the cysteine protease fraction and unfractionated TSBP, female worm size was lower (Table 2). A couple possible explanations for the lack of reduced FECs for recombinant hmcp-1, 4 and 6/Quil-A might be immunization with unpurified *E. coli* cell extracts and/or expression as GST-fusions, of which the GST tag was shown to account for nearly 80% of the total serum IgG response in immunized lambs (Redmond and Knox, 2004). Another possible explanation for the observed reductions in worm burdens but not FECs might be due to the fewer number of worms having elevated fecundities (eggs/adult female), which could be a possible compensation. Either way, these findings clearly indicate that the intestinal TSBP cysteine proteases are partially protective hidden antigens against haemonchosis and hmcp-1, 4 and 6 are candidate immunogens for a recombinant subunit vaccine. However, although there appears to be some trend between protection and serum IgG responses, lack of significant immune correlates indicates the need for further studies of the intestinal TSBP cysteine proteases, as well as optimization in different recombinant expression systems, which should also be extended to the H11 aminopeptidase and H-gal-GP complexes.

In a separate study, another thiol-binding fraction of *H. contortus* adult ES enriched with cysteine and metalloprotease activity was formulated with Alhydrogel and immunized three times s.c. at 15 *µ*g doses into Zwart-Bles lambs (Bakker *et al.* 2004). The thiol-binding fraction of ES induced elevated ES-specific serum IgG, IgA and IgE, but did not significantly elevate mucosal antibody and lymphoproliferative responses. These immunizations resulted in an average 52 and 50% lower *H. contortus* FECs and adult worm burdens, respectively, compared with Alhydrogel alone, but these results were not significant. Although the lower FECs and adult worm burdens were not statistically significant, fecundity was significantly reduced for the thiol-binding fraction, while not for the unbound fraction or, surprisingly, total ES, injected at 60 and 75 *µ*g doses, respectively, and all formulated with Alhydrogel. It was speculated that the choice of adjuvant, although good for thiol-binding fractions, might not have been good for total ES products and thus led to the poor vaccine efficacy observed (Bakker *et al.* 2004). There are no published data on the vaccine efficacy of recombinant ‘ES-thiol’ proteins against haemonchosis.

To date, the combination of native H11 and H-gal-GP gut membrane complexes are the most protective antigen cocktail against haemonchosis. As mentioned above, the commercialized *H. contortus* vaccine Barbervax includes these native complexes and is highly effective at reducing worm burdens and egg counts below troublesome levels. A major issue though is that a protective memory response is not induced in vaccinated lambs during challenge infection, so repeated immunizations are required. It is not yet clear why a protective memory response fails to be induced during challenge infection, but it might be due to the complexes containing exclusively hidden antigens.

#### Ostertagia ostertagi

*Ostertagia ostertagi* is a major trichostrongyloid of cattle that infects the abomasal mucosa feeding on mucus and mucosal tissue, but unlike *H. contortus*, is not an obligate blood feeder (Table 1). These differences in feeding habit likely reflect the contrasting data found in a vaccine efficacy study that included the H-gal-GP fraction, already detailed above and O-gal-GP fraction, both formulated with Quil-A, against haemonchosis and ostertagiasis, respectively (Smith *et al.* 2000). While H-gal-GP/Quil-A was again found to be highly protective against haemonchosis at 100 *µ*g doses in Suffolk–Greyface cross-lambs, immunization of MontBéliard calves with O-gal-GP/Quil-A at 200 *µ*g doses failed to provide consistent protection against ostertagiasis. Quite amazingly though, immunization of O-gal-GP/Quil-A into lambs cross-protected against *H. contortus* with 81–97% and 57–84% lower FECs and worm burdens, respectively, compared with Quil-A alone. Thus, without sufficient blood ingestion by *O. ostertagi*, host antibodies are most likely incapable of binding to and blocking/neutralizing O-gal-GP within the intestine unlike for *H. contortus*, indicating that differences in feeding habits can yield very contrasting results with the same vaccine (Smith *et al.* 2000). These data also support the notion that blocking/neutralizing antibodies may be a good strategy for vaccine-induced protection against blood-feeding STHs.

An *O. ostertagi* adult ES-thiol fraction enriched with cysteine protease activity, but with the most abundant antigens being activation-associated secreted proteins 1 (*Oo*-ASP-1) and 2 (*Oo*-ASP-2), as well as (separately) a membrane-bound thiol subfraction similar to TSBP above, were formulated with Quil-A and immunized three times i.m. at 100 *µ*g doses into MontBéliard calves (Geldhof *et al.* 2002, 2003). Interestingly, the membrane-bound thiol subfraction, which, considering its homology to TSBP, is most likely intestinal, failed to protect against *O. ostertagi* challenge, consistent with the contrasting results with H-gal-GP and O-gal-GP mentioned above. However, immunization with the ES-thiol fraction resulted in an average 60% lower FECs, 18% lower worm burdens (though not significant) and significantly smaller worms compared with Quil-A alone. Protection was correlated with the levels of IgA, IgG1 and IgG2 in the abomasal mucus, and the numbers of infiltrated eosinophils and mast cells within the abomasal mucosa were also slightly correlated with protection (Geldhof *et al.* 2002). In a follow up study (Geldhof *et al.* 2004), the same ES-thiol fraction was formulated with either Quil-A or Alhydrogel, and ES-thiol/Quil-A again resulted in similar levels of protection, while ES-thiol/Alhydrogel failed to protect. ES-thiol/Quil-A resulted in elevated IgA, IgG1 and IgG2 levels in the abomasal mucus, and elevated numbers of eosinophils, mast cells and ‘global leucocytes’ in the abomasal mucosa compared with ES-thiol/Alhydrogel. These findings indicated that Quil-A induces stronger mucosal antibody and cellular immune responses to ES-thiol compared with Alhydrogel, which likely accounts for the superior protection (Geldhof *et al.* 2004). Taken together, the data suggest that for blood-feeding STHs, blocking/neutralizing serum antibody responses may be more important whereas for non-blood feeding STHs, it might be mucosal antibody and cellular immune responses that are more important.

It was later shown that immunization of cattle with three different subfractions of ES-thiol (ASP-enriched, cysteine protease-enriched and the remaining non-cysteine protease-enriched or ‘rest’ subfraction) all resulted in even lower *O. ostertagi* FECs compared with ES-thiol (Meyvis *et al.* 2007). Thus, proteins within all three ES-thiol subfractions have protective capacity against ostertagiasis. Unfortunately, data for recombinant (r)*Oo*-ASP-1 alone, a dominant component in ES-thiol subfractionation, are not as promising. r*Oo*-ASP-1 was expressed in baculovirus-insect cell expression system, the cell extract was formulated with Quil-A and injected three times i.m. at 100 *µ*g doses into cattle, and no differences in *O. ostertagi* FECs or worm burdens were observed (Table 2) (Geldhof *et al.* 2008). Serum from r*Oo*-ASP-1/Quil-A resulted in much weaker general antibody responses to a native *Oo*-ASP-1/2-enriched fraction compared with serum from cattle immunized with the native *Oo*-ASP-1/2-enriched fraction (ODs of ~0·3 and ~2·1, respectively) (Table 2). It was proposed that r*Oo*-ASP-1 was incorrectly folded or lacked the protective post-translational modifications, or that r*Oo*-ASP-1 alone may be insufficient for protection possibly requiring the addition of r*Oo*-ASP-2 (Geldhof *et al.* 2008). However, there was no mention of the possibility that purification of r*Oo*-ASP-1 from the crude insect cell extract may improve vaccine efficacy, similar to that mentioned above for bac-r*Hc*-H11.

Native *O. ostertagi* polyprotein allergen (*Oo*-PA) formulated with Quil-A resulted in ~60% lower *O. ostertagi* FECs with protection correlated with mucosal IgG1 and IgG2 responses, while as with r*Oo*-ASP-1, recombinant protein (this time made in *E. coli*) completely failed to protect (Table 2) (Vercauteren *et al.* 2004). Thus, although recombinant forms of protective *O. ostertagi* antigens have failed to protect cattle from challenge infections, optimization of and/or use of alternative expression systems and/or optimized purification are warranted in order to conclusively determine their vaccine efficacy.

#### Teladorsagia circumcincta

*Teladorsagia circumcincta* is another abomasal mucus dweller very similar to *O. ostertagi*, but mostly infects sheep (Table 1) (Zajac, 2006). Eight different antigens either, (1) naturally immunodominant for IgA [cathepsin F-1 (*Tci*-CF-1); astacin-like metalloproteinase-1 (*Tci*-MEP-1); 20 kDa protein of unknown function (*Tci*-ES20); and *Tci*-ASP-1]; (2) homologous to protective antigens from other strongylids (*Tci*-SAA-1); or (3) potential immunomodulatory proteins [macrophage migration inhibitory factor-1 (*Tci*-MIF-1); calcium-dependent apyrase-1 (*Tci*-APY-1); and transforming growth factor β homologue (*Tci*-TGH)], were evaluated for efficacy in a single, recombinant multivalent vaccine. Recombinant proteins were either expressed in *E. coli* (r*Tci*-MEP-1; r*Tci*-ASP-1; r*Tci*-SAA-1; r*Tci*-MIF-1; r*Tci*-APY-1; and r*Tci*-TGH) or *P. pastoris* (r*Tci*-CF-1 and r*Tci*-ES20), formulated with Quil-A, and injected three times s.c. at 50 *µ*g doses each into Texel cross-bred lambs. Immunization resulted in elevated serum and abomasal mucosal IgG and IgA responses for nearly all recombinant immunogens, and 70% lower *T. circumcincta* FECs and 55% lower worm burdens compared with Quil-A alone (Table 2) (Nisbet *et al.* 2013). Thus, this study provided proof of concept that recombinant subunit vaccines can work against trichostrongyloids of livestock. However, whether individual components of the recombinant vaccine cocktail can protect against teladorsagiasis, or whether specific combinations are required has not been resolved.

## RECOMBINANT SUBUNIT VACCINES FOR ASCARIDS

Ascarids (Nematoda clade III, family Ascarididae) are serious parasites of humans (*Ascaris lumbricoides*), pigs (*Ascaris suum*), dogs (*Toxocara canis*) and cats (*Toxocara cati*) (Table 1). Species of the *Baylisascaris* genus are also important parasites of wild animals such as giant pandas (*Baylisascaris schroederi*) and raccoons (*Baylisascaris procyonis*), of which the latter can cause lethal zoonoses. *A. lumbricoides*, the human giant roundworm (can be up to 50 cm in length), infects over 800 million human beings, making it the most common of the human STHs (Pullan *et al.* 2014). Embryonated ascarid eggs, which can persist in the soil for over 10 years, are ingested and hatch within the small intestine, where the infectious larvae burrow into the intestinal mucosa entering the bloodstream, and then pass through the liver and heart ultimately breaking free in the pulmonary alveoli where the larvae undergo maturation. Similar to *N. americanus*, larvae migrate up the bronchial tree into the throat, and are then swallowed eventually reaching the small intestine once again where they develop into adult worms. Adult females can produce over 200 000 eggs per day, and these worms can live within the small intestine for years (Crompton, 2001). Infection by *A. lumbricoides* (and *A. suum*) is relatively asymptomatic as this nematode consumes luminal contents rather than host tissue, yet stunts physical and cognitive development in children due to significant malnutrition (Stephenson *et al.* 2000). On the other hand, larval migration can lead to various pulmonary pathologies, and heavy patent infections can cause life threatening gut obstructions, and obstructions to biliary and pancreatic ducts due to occasional upstream migration. *Baylisascaris procyonis* infection is particularly and highly dangerous if eggs are ingested by humans due to the potential for larvae to migrate to the brain. Also, severe symptoms in young dogs and cats result from infection by *T. canis* and *T. cati*, respectively, and if humans are infected by them syndromes known as visceral and ocular larva migrans can develop where larvae migrate to the heart, brain or eye, of which the former can be fatal. Thus, preventing infections by ascarid parasites is critical (Crompton, 2001).

Currently there are no published results for recombinant subunit vaccines against *A. lumbricoides*, while several studies have been published for *A. suum* and *B. schroederi*. Given the recent common ancestries between these ascarid species and *A. lumbricoides*, such studies are highly relevant to this most prevalent human STH.

### Ascaris suum

#### Intranasal immunization with cholera toxin B (CTB)subunit

*Ascaris suum* 14 and 16-kDa proteins (*As*14 and *As*16), as well as *As*24 and *As*37 mentioned below, are conserved in clade III helminths, and were suspected to be highly antigenic as they were determined to be abundantly expressed on the surface of the infective L3 stage (Kasuga-Aoki *et al.* 2001) and are present in ES products. Both *As*14 and *As*16 are homologous to *Ac*-16 mentioned above (Fujiwara *et al.* 2007).

r*As*14 was expressed in *E. coli*, conjugated to CTB subunit, an adjuvant that enhances mucosal and systemic immune responses, and immunized i.n. into BALB/c mice at a primary dose of 50 *µ*g with two boosters of 30 and 10 *µ*g (Tsuji *et al.* 2001). Mice immunized with r*As*14-CTB had significant mucosal IgA and serum IgG (with IgG1 the highest) and IgE responses, and resulted in 60% lower larval *A. suum* worm burdens in the lungs compared with both CTB and r*As*14 alone (Table 2) (Tsuji *et al.* 2001).

r*As*16 was also expressed in *E. coli* and conjugated to CTB, and was immunized three times i.n. into BALB/c mice with a primary dose of 25 *µ*g and two boosters of 15 and 10 *µ*g in one study (Tsuji *et al.* 2003) and into cross-bred Landrace × Whitelarge × Duroc piglets at 300 *µ*g in another study (Tsuji *et al.* 2004). In the former study, mice immunized with r*As*16-CTB had significant serum IgG (with equal contributions from IgG1, IgG2a and IgG3) and IgE responses, but an insignificant mucosal IgA response, and highly elevated splenic r*As*16-stimulated IFN-γ and IL-2, as well as significant IL-10, but not IL-4 (Table 2) (Tsuji *et al.* 2003). Somewhat differently in the latter study, piglets immunized with r*As*16-CTB had significant mucosal IgA and serum IgG (with IgG1 the highest) (IgE responses were not evaluated), and was also shown to stimulate elevated IFN-γ, IL-4 and IL-10 from PBMCs (Table 2) (Tsuji *et al.* 2004). IL-4 and IL-10 were more highly elevated than IFN-γ in the piglets (Table 2). Both mice and piglets immunized with r*As*16-CTB, similar to r*As*14-CTB above, had 60% lower larval worm burdens in the lungs compared with both CTB and r*As*16 alone (Table 2) (Tsuji *et al.* 2003, 2004).

Thus, both r*As*14-CTB and r*As*16-CTB induced protection against *A. suum* larval colonization of the lung in association with mixed T_H_1/T_H_2 mucosal and/or systemic responses, of which such protection was completely dependent upon CTB conjugation. Also, anti-r*As*16-CTB mouse and piglet serum localized *As*16 to the worm hypodermis and intestine (Tsuji *et al.* 2003, 2004) and inhibited moulting of *A. suum* L3 *in vitro* (Tsuji *et al.* 2004). It was speculated that the mechanism of r*As*16-CTB-induced immunity involves induction of antibodies that block *As*16, resulting in moulting defects.

#### Parenteral immunization

r*As*24 was expressed in *E. coli*, formulated with CFA, and injected three times s.c. at 50 *µ*g doses into BALB/c mice (Islam *et al.* 2005*a*). Immunization of mice with r*As*24/CFA resulted in highly significant serum IgG1, IgG2a and IgG2b responses, significant r*As*24-stimulated splenic IFN-γ and IL-10 (but not IL-4) responses, and a significant 58% less larvae recovered from the lungs compared with CFA alone (Table 2). This study clearly indicated an association between r*As*24/CFA-induced protection and a mixed T_H_1/T_H_2-type immune response. The finding that anti-r*As*24 serum IgG obtained from immunized mice inhibited the L3 to L4 moult by 26% *in vitro* suggests a role for serum IgG in r*As*24/CFA-induced protection. *As*24 was also strongly localized to the hypodermis, supporting a potential role in the moulting process (Islam *et al.* 2005*a*).

*As*37, in addition to being abundantly expressed on the surface of *A. suum* L3, is immunodominant and recognized by serum obtained from previously infected, fenbendazole-treated pigs (Kasuga-Aoki *et al.* 2001). The *As*37 amino acid sequence harbours multiple Ig domains and has similarity to proteins of the Ig superfamily, as well as nematode twitchin and mammalian skeletal muscle titin. Native *As*37 was localized in the muscle and hypodermis, as well as in the ovaries, eggs and lateral and dorsal chords of *A. suum* adult females, indicating a rather ubiquitous expression pattern. Sera from rabbits, mice and pigs infected with *A. suum* embryonated eggs reacted with *E. coli-*expressed r*As*37, supporting natural immunogenicity of *As*37. BALB/c mice were injected with r*As*37/CFA three times s.c. (doses not reported), and, although immunized mice had significant serum IgG responses, significant reduction in the number of larvae in the lungs was not observed (Table 2). It was proposed that alternative expression systems and/or routes of immunization need to be tested in order to determine whether or not r*As*37 has protective vaccine efficacy (Tsuji *et al.* 2002).

*Ascaris suum* inorganic pyrophosphatase (*As*-PPase) is conserved in ascarids and was shown to play a role in larval development and moulting, but was also shown to be expressed throughout the life cycle, and to localize in the hypodermis and adult reproductive tissues (Islam *et al.* 2003). Knockdown of *As*-*PPase* by RNA interference (RNAi) inhibited moulting of L3 to L4 *in vitro* by 31%, with only around 56% reduction in *As*-PPase protein levels (Islam *et al.* 2005*b*). r*As*-PPase was expressed in *E. coli*, formulated with TiterMax Gold adjuvant, and injected three times s.c. at 50 *µ*g doses into BALB/c mice. Immunization with r*As*-PPase/TiterMax resulted in significant serum IgG responses (predominantly IgG1), significant production of splenic IL-10 (but not IFN-γ, IL-2 or IL-4) after r*As*-PPase stimulation, and over 70% less *A. suum* larvae in the lungs compared with TiterMax alone (Table 2). Also, anti-r*As*-PPase serum from immunized mice recognized native *As*-PPase, localized it to the hypodermis of lung-stage larvae, and inhibited L3 to L4 moulting *in vitro* by up to 57%. Finally, immunoreactive plasmids from the Novotope system mapped IgG-binding epitopes to sites spread across the whole r*As*-PPase protein, including the active site (Islam *et al.* 2005*b*). Although the latter experiment did determine that epitopes overlap with the active site, the Novotope system is limited to contiguous epitopes, and it was not resolved for whether or not protection is mediated by serum IgG-binding to linear or discontinuous conformational epitopes within the *As*-PPase active site. Nonetheless, this study revealed r*As*-PPase/TiterMax Gold as the recombinant subunit vaccine that provides the greatest protection against invasive L3 tissue migration to date in mice. It was speculated that the mechanism of vaccine-induced immunity involves IgG-binding and blocking/neutralizing native *As*-PPase in the hypodermis, thus disrupting larval development and moulting, and hence, efficient migration and development within the lung (Islam *et al.* 2005*b*). Surprisingly, the protection was clearly associated with IL-10, an anti-inflammatory cytokine (Couper *et al.* 2008).

Enolase is suspected to be involved in glycolysis, and knockdown of *A. suum enolase* (*AsEnol*) by RNAi indicated an important role in larval development (Chen *et al.* 2011). *As*Enol has been identified in ES products, despite the fact that it is missing an N-terminal signal peptide (Huang *et al.* 2008), suggesting a function in the host. Chen *et al.* (2012) investigated the vaccine efficacy of *As*Enol using a DNA vaccine approach in mice. An *AsEnol* cDNA was cloned into the pVEX I expression plasmid, transfected into Marc-145 cells and expression and immunogenicity of r*As*Enol was verified by indirect immunofluorescence assay using pVEX I-*AsEnol* serum from mice. pVEX I-*AsEnol* was immunized three times i.m. at 100 *µ*g doses into Kunming mice in the absence of a transfection reagent or adjuvant. Immunization resulted in significant total serum IgG responses against *A. suum* crude larval extract (*As*CE), and significant splenic *As*CE-stimulated IFN-γ, IL-2, IL-4 and IL-10 responses (Table 2). IFN-γ was much more highly elevated compared with the other cytokines. Also, immunization with pVEX I-*AsEnol* resulted in significant proliferative responses in splenocytes stimulated with *As*CE (Table 2), indicating an established memory T_H_ cell response. Finally, pVEX I-*AsEnol* resulted in an average 44% less larvae recovered in either the liver or lung compared with pVEX I alone (Table 2), thus indicating significant efficacy of this DNA vaccine, characterized by a T_H_1-skewed cytokine profile with minor T_H_2 contributions (Chen *et al.* 2012).

### Baylisascaris schroederi

*Baylisascaris schroederi* antigens (Ag) 1, 2 and 3 are homologous to *As*14, *As*16 and *As*37 mentioned above, respectively. Hence, their efficacies as recombinant subunit vaccines against experimental *B. schroederi* infection in mice were evaluated in order to develop alternative control measures for this critical STH of the endangered giant panda, of which the prevalence of infection is between 70 and 100% (Wang *et al.* 2008; He *et al.* 2009, 2012). r*Bs*-Ag1 (He *et al.* 2012), r*Bs*-Ag2 (He *et al.* 2009) and r*Bs*-Ag3 (Wang *et al.* 2008) were expressed in *E. coli*, formulated with CFA, and injected three times i.p. at 30 *µ*g doses into BALB/c mice. Mice immunized with all three vaccines had significant serum IgG responses and had a significant 60% less *B. schroederi* larvae recovered in the lungs compared with CFA alone (Table 2) (Wang *et al.* 2008; He *et al.* 2009, 2012). These studies were in concordance with the vaccine studies for r*As*14 and r*As*16, but different from the results obtained with r*As*37, which may reflect the differences in route of immunization (s.c. *vs* i.p.) or other technical/biological factors. Regardless, these three recombinant immunogens serve as possible candidates for a vaccine to control baylisascariasis in giant pandas.

The efficacy of the *B. schroederi* homologue of *As*-PPase mentioned above (*Bs*-PYP-1) as a recombinant subunit vaccine against experimental infection in mice was also investigated (Xie *et al.* 2013). r*Bs*-PYP-1 was expressed in *E. coli*, formulated with CFA, and injected three times s.c. at 50 *µ*g doses into BALB/c mice in two separate trials. In both trials, immunization with r*Bs*-PYP-1/CFA resulted in significant serum IgG responses (with IgG1 much greater than IgG2a), significant production of r*Bs*-PYP-1-stimulated splenic IL-4 and IL-10 (with IL-10 much greater than IL-4), without production of IFN-γ or IL-2, and a significant 70% less *B. schroederi* larvae recovered in the lungs (Table 2). Other than the significant production of IL-4, these results were in concordance with the findings for the r*As*-PPase homologue mentioned above, again demonstrating a unique role for IL-10 in vaccine-induced immunity to these ascarids. Also similar to *As*-PPase, native *Bs*-PYP-1 was localized to the hypodermis and reproductive tissues with anti-r*Bs*-PYP-1 serum, but also localized to the muscle tissues and intestine, and thus, was more ubiquitously expressed (Xie *et al.* 2013). The inorganic pyrophosphatase conserved in ascarids therefore remains as the most promising vaccine candidate to date for these critical parasites of animals and humans.

It must be mentioned that all of these previous studies of recombinant subunit vaccines in ascarids have focused on target antigens abundantly expressed and/or present on the surface of infective L3. Although there is no direct evidence for pre-existing serum IgE against ascarid L3 antigens resulting in allergic reactions when immunized into previously infected humans or animals, the studies in hookworms present an alarming issue. Studies must be conducted to determine if these candidate immunogens will pass safety testing in humans and veterinary animals, because if not, future developments in ascarids should no longer focus on L3 antigens.

## RECOMBINANT SUBUNIT VACCINES FOR TRICHURIDS

Trichurids (*Trichuris* spp.; whipworms; Nematoda clade I) are critical parasites of livestock, dogs, cats and humans, and infect various rodents. *Trichuris trichiura* (Table 1) is the only well-known human trichurid, infecting almost 500 million people (Pullan *et al.* 2014). Trichuriasis can be characterized by painful passage of the stool and rectal prolapse with heavy infections, and in children, can lead to severe anaemia, stunted growth and cognitive development. Trichurids begin their life cycle when embryonated eggs are ingested by the host. Once the eggs reach the duodenum, they hatch. Larvae either mature in the small intestine, or travel to the cecum of the large intestine for maturation, feeding on tissue fluids, the mucosal epithelium, and possibly blood, but evidence for the latter remains inconclusive. Adult trichurids anteriorly fixed within the intestinal mucosa mate, and each female lays between 3000 and 20 000 unembryonated eggs per day in the cecum and ascending colon that are passed with the feces, embryonating after several weeks and continuing the next cycle once ingested. The lifespan of *T. trichiura* is about 1 year (Bundy and Cooper, 1989).

Although there are no published studies for recombinant subunit vaccines for any trichurid, vaccination of mice with adult *Trichuris muris* ES products resulted in almost complete protection against *T. muris* egg challenge, in association with a classical T_H_2 response (Dixon *et al.* 2010). Also, a few studies have obtained promising results for the zoonotic parasite *Trichinella spiralis* (Table 1), which, like trichurids, is also found in clade I Nematoda. Although *T. spiralis* has a considerably different life cycle than trichurids – transmission to humans occurring from ingestion of larvae-contaminated meat, followed by larval maturation to adults that deposit eggs within the small intestine that hatch, and then the larvae penetrate the intestinal mucosa and subsequently migrate to muscle tissue for maturation into cysts – many antigens are likely to contain high amino acid identities and to potentially have similar functions.

Three related studies in mice determined the vaccine efficacy of the immunodominant and/or immunomodulatory surface and/or ES proteins *Ts*87, *Ts*Gp43 and paramyosin (*Ts*Pmy) delivered as DNA vaccines in recombinant, attenuated *Salmonella enterica* serovar Typhimurium for induction of mucosal immune responses (Yang *et al.* 2010*b*; Pompa-Mera *et al.* 2011; Wang *et al.* 2016). Vaccines were administered three times either orally or i.n. and elicited significant mucosal IgA and/or serum IgG1/IgG2a as well as elevated T_H_1 and T_H_2-associated cytokines (Table 2). Vaccination resulted in significantly less (between 30 and 50%) *T. spiralis* worm burdens in muscle tissue compared with control (Table 2). Though these recombinant subunit, oral vaccines for *T. spiralis* are encouraging for trichurids, the efficacy of oral vaccination against *T. muris* in mice was shown to depend upon host genetics, and less so for parenteral vaccination (Robinson *et al.* 1995). Nonetheless, *Ts*Pmy resulted in similar vaccine efficacy when administered s.c. as an *E. coli*-expressed protein-based vaccine formulated with CFA (Yang *et al.* 2010*a*), and thus, appears to be the most encouraging of the three antigen targets for potential application to trichurids.

A very recent study (Xu *et al.* 2017) used a naked DNA vaccine approach (i.e. no adjuvant or transfection reagent) to test the vaccine efficacy of a serine protease (*Ts*-NBLsp) expressed specifically in the newborn larval (NBL) stage, which is the stage when *T. spiralis* colonizes the striated muscle tissue after migrating from the small intestine. A *Ts-NBL*sp cDNA was cloned into pcDNA3·1+ and injected twice i.m. at 60 *µ*g doses into the quadriceps of Kunming mice, where expression of the recombinant *Ts*-NBLsp protein was confirmed by immunofluorescence. Mice immunized with pcDNA3·1(+)-*Ts-NBL*sp had elevated serum IgG1 and IgG2a responses, with the latter higher than the former, but responses were weak overall (maximum IgG1 OD450 = ~0·3; maximum IgG2a OD450 = ~0·55; background = ~0·1; 1 : 50 serum dilution) (Table 2), consistent with the previous studies on DNA vaccines mentioned above. Interestingly, the ratio of CD4^+^/CD8^+^ T cells in peripheral blood was significantly lower in both pcDNA3·1+/*Ts-NBL*sp and pcDNA3·1+ empty vector control mice compared with PBS control mice (i.e. the population of CD4^+^ T cells were reduced and/or CD8^+^ T cells expanded) (Table 2). Consistent with an expansion of CD8^+^ T cells in peripheral blood, IFN-γ was also significantly elevated in the peripheral blood for both pcDNA3·1+/*Ts-NBL*sp and pcDNA3·1+ empty vector control mice compared with PBS control mice, and higher (but not significant) in pcDNA3·1+/*Ts-NBL*sp compared with pcDNA3·1+ (Table 2). On the other hand, IL-10 and IL-4 were significantly elevated in the peripheral blood of only pcDNA3·1+/*Ts-NBL*sp mice (Table 2). Mice immunized with pcDNA3·1+/*Ts-NBL*sp had ~78% less *T. spiralis* larvae recovered in muscle tissue (Table 2), and interestingly, pcDNA3·1+ empty vector control mice also had a significant 30% less *T. spiralis* larvae, compared with PBS control mice (Xu *et al.* 2017). These findings indicated that: (1) the pcDNA3·1+ vector alone induces a non-specific cell-mediated immune response within the muscle tissue that reduces susceptibility to *T. spiralis* larval colonization, and (2) pcDNA3·1+/*Ts-NBL*sp is a highly protective DNA vaccine that greatly reduces NBL colonization of the muscle tissue, in association with a mixed T_H_1/T_H_2-type immune response. It is also worth mentioning that the apparent elevation in CD8^+^ T cells, and association with protection, makes sense as T_H_1 effectors are known to be protective against the *T. spiralis* NBL stage (but not T_H_2 effectors), which becomes an intracellular pathogen within the muscle tissue. Finally, although *T. spiralis* infects its host much differently, it would be worthwhile to explore the NBLsp homologue in trichurids to determine if by targeting this antigen, similarly high levels of protection can be achieved.

## DISCUSSION

These previous studies of recombinant subunit vaccines for STHs have reported many possible candidates for further optimization, and some that progressed to clinical trials. However, collectively reviewing all of these studies has revealed many differences in vaccine design and their administration. Examples include the use of protein or DNA vaccines, *E. coli* or eukaryotic expression systems, T_H_1 or T_H_2-biased adjuvants, many different routes of immunization, different amounts of immunogen and the number of injections, among many other inconsistencies. Below, we briefly discuss and interpret the results from these previous studies as a whole in an attempt to help guide future developments.

### Protein or *DNA vaccines?*

An important difference between protein and DNA vaccines, is that the former has been used successfully in humans since the mid-20th century (Plotkin, 2014), but not a single DNA vaccine has been shown to work in humans. An advantage of protein vaccines is that the immunogen (i.e. an extracellular antigen) is processed and presented on Class II MHC by professional APCs, and along with adjuvant, this can shape intricate T_H_ responses, and clearly the majority of successes with recombinant subunit vaccines for STHs have been protein-based. However, with DNA (and RNA) vaccines the immunogen must be translated within the host cell cytoplasm, and hence, can only be presented by Class I MHC. Thus, the immune response(s) is generally limited to T_H_1, and particularly to CD8^+^ T_C_ cells (Ferraro *et al.* 2011). This may explain the weak antibody responses generated against r*Acey*-MEP-6 and r*Acey*-MEP-7 in golden hamsters using the pcDNA3·1+/Fugene 6 system against *A. ceylanicum* (Wisniewski *et al.* 2013, 2016), against r*Ss*-EAT-6 peptide in mice using the Lambda Uni-zap XR vector/*GM-CSF* system against *S. stercoralis* (Kerepesi *et al.* 2005), and against r*Ts*-NBLsp in mice using pcDNA3·1+ alone against *T. spiralis*. However, DNA vaccines in mice have proven to be highly efficacious against *A. suum* (Chen *et al.* 2012) and *T. spiralis* (Yang *et al.* 2010*a*; Pompa-Mera *et al.* 2011; Wang *et al.* 2016), with significant immunogenicity (albeit relatively weak for pcDNA3·1+/*Ts-NBLsp*) and protection achieved. Moreover, though DNA vaccines against *A. suum* and *T. spiralis* resulted in significant T_H_1 responses, as would be expected, there were contributions from T_H_2 cytokines as well. These studies demonstrate that the immune responses to DNA vaccines against STHs are likely to be more complex than that appreciated, and this type of recombinant subunit vaccine may prove to be broadly applicable in the future with more thorough studies. However, it will be important for future studies to address the safety concerns of host gene modification and uncontrollable gene expression before DNA vaccines can be a realistic STH control measure.

### Escherichia coli or eukaryotic expression systems for protein-based vaccines?

*Escherichia coli* would be the most cost effective system for expressing recombinant proteins for STH vaccines, which is important given that the target populations are people living in poverty. However, it is widely known that recombinant expression of eukaryotic proteins in *E. coli* often results in the proteins being expressed in insoluble inclusion bodies. Inclusion bodies must then be solubilized with harsh detergents, and often reduced, thus causing denaturation with the major challenge of refolding into native confirmation. Also, even in the rare event that the protein does express in *E. coli* in the soluble fraction, still, folding is often incorrect, and eukaryotic post-translational modifications – common on secreted proteins – are deficient. These deficiencies of *E. coli* are significant for vaccine development given that adequate immunogenicity often requires discontinuous conformational B cell epitopes (Andersen *et al.* 2006). Hence, eukaryotic expression systems such as yeast (*P. pastoris* or *K. lactis*) and baculovirus-insect cells are likely to be more suitable for production of recombinant proteins for vaccines against STHs. On the other hand, although the majority of successful recombinant subunit vaccines for the strongylids have been with these eukaryotic expression systems, with most attempts having failed with *E. coli* (especially in the trichostrongyloids), the major successes in ascarids with *E. coli* presents a significant incongruence. Could it be that some immunogens (e.g. those mentioned above for ascarids) induce protection through linear T cell epitopes independent from B cells, similar to the natural immune responses (Anthony *et al.* 2007; Nair and De'Broski, 2016), while other immunogens (e.g. many of those mentioned above for stongylids) require non-linear B cell epitopes? It seems likely that success in any expression system will vary on a protein-to-protein basis for any STH, and more thorough studies of the immunobiology of STH infections will help to explain the protein-to-protein variations that have been observed.

### What are the best immune correlates of vaccine-induced protection?

#### B cells

There have been many immune factors found to be associated with vaccine-induced protection against STHs. Serum IgG1, IgG2 and IgE, and mucosal IgA [i.e. secretory (s)IgA], IgG1 and IgG2 are antibodies often found to be elevated in immunized animals. IgG1/IgG2 in both serum and mucosa have been found to be correlated with the level of protection, and in some cases, so has sIgA. In some cases, equal balance of IgG1 and IgG2 were found to correlate with the greatest level of protection. As IgG1 is considered the T_H_2 subclass, while IgG2 is considered the T_H_1 subclass, most relate this equal balance to a mixed T_H_1/T_H_2 response. However, although T_H_2-dominated cytokine profiles were nearly always associated with IgG1-dominated serum antibody, many of the previous studies report IFN-γ-dominated cytokine profiles also with highly elevated IgG1, often higher than IgG2. So it is unclear if IgG1 is specific for T_H_2 in vaccine-induced immune responses to STHs, and cytokines must also be evaluated to determine the type of T_H_ response. Also, in some studies protection was correlated with IgG1 or balanced IgG1/IgG2, while in others, sIgA was more highly correlated. Importantly, although the IgG class has been ascribed the function of ‘blocking/neutralizing’ target antigen function for hookworms, inhibiting protein activity *in vitro*, passive transfer experiments with purified antibodies have not been performed. Thus, it has not been conclusively determined if antibodies in general are sufficient for vaccine-induced protection, and if any one class is sufficient. However, it was shown that passive transfer of serum specific for r*Ss*-IR resulted in similar levels of protection against *Ss* challenge infection compared with immunized donor mice (Abraham *et al.* 2011), thus supporting the role of antibodies in vaccine-induced protection. That said, Abraham *et al.* (2011) speculated that antibodies were not blocking/neutralizing antigen function *per se*, but that the protection was *via* complement and/or ADCC.

#### T cells

Antigen recall responses in peripheral blood, spleens, or mLNs of immunized animals have found that IFN-γ, IL-4 and IL-13 are frequently elevated indicating that protection is often associated with mixed T_H_1/T_H_2 responses, consistent with common elevations of both IgG1 and IgG2. In some cases, IL-10 was also elevated, and in other cases, IL-10 dominated the cytokine pool, thus demonstrating that IL-10s role in vaccine-induced immunity to STHs may be more complicated than typically appreciated (i.e. it may not be just an anti-inflammatory cytokine). But the levels of all these cytokines were highly variable between studies. As with antibodies, it is unclear which, if any, of these cytokines are necessary for vaccine-induced protection. *In vivo* experiments with cytokine knockout animals or with blocking/neutralizing antibodies will need to be performed to determine if any of these cytokines are necessary for protection, and adoptive transfer experiments should be performed to determine whether T cells are sufficient, or whether antibodies are also required. Furthermore, many more cytokines should be evaluated in future studies to more thoroughly characterize the T_H_ responses associated with protection. Finally, studies should compare the levels of cytokines produced by lymphocytes in response to antigen stimulation in the different lymph tissues. Although most studies have found that lymphocytes from immunized animals proliferate significantly in response to antigen, it would also be worthwhile to compare proliferative responses between different lymph tissues.

#### Innate immune cells

Studies of the trichostrongyloids showed nicely that the granulocytes eosinophils, basophils and mast cells were elevated locally within the abomasum, and in some cases correlated with the level of protection. Such experiments should be extended to the other STHs to determine whether these innate immune cells, or others, such as neutrophils, macrophages, dendritic cells and even natural killer cells, are (or are not) significantly elevated within the local tissue, which could ultimately help to enhance vaccine-induced protection.

### Does adjuvant really matter for parenteral vaccines?

One of the most variable factors among these previous studies was the choice of adjuvant. Given the conserved association of T_H_2-type responses with immunity to STHs, T_H_2-biased adjuvants make sense. However, the paradigmatic natural immune responses to STHs are clearly not conserved with the immune responses associated with recombinant subunit vaccines. Loukas *et al.* (2004) showed that whether T_H_1 or T_H_2-biased adjuvant was used with r*Ac*-CP-2 did not significantly change the immunogenicity and protection against *A. caninum* challenge infection induced in beagles. And multiple studies for hookworms used either T_H_1 or T_H_2 adjuvants with success, and the associated immune responses – elevated IgG1/IgG2 – were overall consistent with both classes of adjuvants (although cytokines were never evaluated for T_H_2 adjuvants, so they cannot be compared with the cytokine changes observed for T_H_1 adjuvants). Also, studies in ascarids and *T. spiralis* had much success with CFA, a heavily T_H_1-biased adjuvant, with elevated, and in some cases dominant, T_H_2 cytokine profiles. It seems reasonable to question if the immunogens alone can override adjuvant induction of T_H_1 and/or T_H_2, but such experiments were not conducted to be certain.

On the other hand, studies in the non-hookworm strongylids found that the choice of adjuvant can determine the outcome of vaccination. Ben Nouir *et al.* (2012) showed that r*Sr*-HSP60, which inherently induces T_H_1, resulted in an IL-13-dominated cytokine profile and partial protection when formulated with alum, but with CFA actually increased susceptibility to *S. ratti* challenge infection. Conversely, in the trichostrongyloids, multiple native immunogens lacked significant protective effects when formulated with T_H_2 adjuvants (IFA or Alhydrogel), but with Quil-A (and CFA) resulted in significant protection (Knox *et al.* 1999; Geldhof *et al.* 2004), possibly suggesting that adjuvant induction of T_H_1 or a mixed T_H_1/T_H_2 response is necessary for the mechanism of vaccine-induced protection, at least in these trichostrongyloids.

Clearly we are far from understanding the mechanism(s) of vaccine-induced protection against STHs, and it is likely that each STH will be distinct in one way or another. Thus, the field of STH vaccinology needs to better bridge with immunobiology for us to more rationally design better recombinant subunit vaccines.

### Parenteral or oral/intranasal immunization?

Parenteral immunizations have dominated the literature for the development of STH vaccines. However, in considering the target populations that are most afflicted by STHs, oral/i.n. vaccines are likely to be more ideal due to their ease of self-administration, which would likely allow for a much wider dispersal, and for their induction of local, mucosal immune responses. Previous studies of *A. suum* and *T. spiralis* had much success with oral or i.n. immunizations, and the success in *A. suum* with simple conjugation of immunogen to CTB (CTB subunit) is promising for all STHs. However, as mentioned above, Robinson *et al.* (1995) showed that BALB/c high and C57BL/10 and B10.BR low-responder mice were all protected against *T. muris* challenge infection when immunized s.c. with *T. muris* adult worm extract, with elevated serum IgG1 and IFN-γ and IL-5 cytokine profiles. However, only BALB/c high and C57BL/10 low-responder mice were protected against *T. muris* when immunized orally, while B10.BR low-responder mice were not protected and had negligible immune responses. Also, the success of oral/gut mucosal vaccines in endemic human populations for other enteric pathogens such as *S. enterica* serovar Typhi and *Vibrio cholera* has been demonstrated to depend largely on underlying genetics (Pasetti *et al.* 2011). Thus, although oral/i.n. vaccines may be a hopeful goal for STH vaccines (especially for humans), the lesser dependence on host genetics for parenteral vaccines suggests a greater likelihood of success in clinical trials.

### Does immunization regimen matter?

A likely underappreciated factor that is important for vaccine design is the number of injections and amount of immunogen per injection. With reductionist vaccine design (at least in the simplest terms), the number of injections and amount of immunogen (as well as route of administration and form of immunogen) can be optimized in order to achieve a desired immune response (e.g. sufficient affinity maturation) (Jardine *et al.* 2016). Nearly all of the previous studies of recombinant subunit vaccines for STHs used three equally dosed injections, with small rodents (mice and golden hamsters) generally receiving between 20 and 50 *µ*g doses, and larger animals (beagles, sheep and cattle) receiving from 50 to 300 *µ*g doses. No studies have actually adequately compared different numbers of injections and doses of the same vaccine, but such a study may prove worthwhile, especially considering that minimizing the number of immunizations (and doses) required for protection will be critical for the success of these vaccines in target populations. A few studies did, however, differ from the standard of three injections, with significant protection achieved with just two injections (Reszka *et al.* 2007; Ben Nouir *et al.* 2012), suggesting that this may be the minimum number. Regardless, it is very likely that the dose and frequency of exposure will vary with antigen and host and will probably have to be empirically determined for each case.

### Should naturally hidden or exposed antigens be targeted?

The mechanisms of vaccine-induced protection against STHs have not been completely resolved, only extensively postulated. To use hookworms and *H. contortus* as an example, the idea behind targeting STH proteins that are present within STHs’ intestinal lumens is that these proteins are accessible to antibodies ingested in the hosts’ blood. The antibodies bind to the target proteins, and block/neutralize their activities/functions. These proteins have been called hidden antigens, as mentioned above, because naturally they are physically concealed from the host immune system inside of the blood-feeding STHs’ bodies. The current definition of hidden antigen does not include those antigens that naturally are physically exposed to the host immune system (e.g. proteins in ES products) and immunologically silent for unknown reasons, which could be distinguished from hidden antigens by from now on being called immune-silent ES proteins. Now hidden intestinal proteins in STHs that do not normally ingest blood (see Table 1) may not be as useful to target, as exemplified by comparing the efficacy of the same vaccine against *H. contortus* and *O. ostertagi* (Smith *et al.* 2000). However, as most non-blood-feeding STHs normally ingest large amounts of mucus within the GI tract, induction of high titre of sIgA as well as IgG within the mucosa might grant these antibodies access to block/neutralize the hidden intestinal proteins within these worms. But again, since passive transfer experiments of purified antibodies specific for individual immunogens have not been performed, it is uncertain whether blocking/neutralizing antibodies are in fact solely responsible for vaccine-induced protection, even against hookworms and *H. contortus*.

Targeting hidden intestinal antigens is justified by studies in hookworms. Naturally immunodominant ASP-2 secreted by hookworm infective L3, although partially protective, resulted in allergic reactions when immunized into previously infected individuals due to pre-existing ASP-2-specific IgE, as mentioned above. Thus, targeting antigens that are not naturally immunogenic avoids such risks. But an important question unaddressed for the hidden antigen approach is whether or not recall responses will be sufficiently induced in memory lymphocytes in vaccinated individuals if in fact the native antigens are hidden from the immune system. In the experimental vaccine studies (including with hidden intestinal antigens), animals were challenged soon after they were immunized, and thus, acute immune responses were likely still existent. In order to answer this important question, it would be worthwhile to at least investigate the lifetime of protection for such vaccines that target hidden intestinal antigens in the animal models for experimental vaccination.

On the other hand, what about targeting antigens exposed on the surface of L4 and/or adult worms (while also absent from L3), or that are secreted into the local environment (i.e. proteins in ES products)? In theory, many of these antigens would be both immunologically accessible and more likely to induce long-lasting recall responses in immunized individuals, because they are in fact exposed. And as long as these antigens are not produced by infective L3 (most notably in hookworms), natural immunogenicity in these later stages may not matter. There would also be the option of using the exposed antigens to direct the natural immune mechanisms that exist to expel STHs from the GI tract. Such an approach may also help to better bridge the currently largely independently operating fields of STH vaccinology and immunobiology.

### How valid are the laboratory challenge infection models?

All of the preclinical vaccine studies above for human STH infections used various laboratory challenge infection models. Although there is no way of conducting preclinical studies directly on humans, there are likely limitations with these models. These limitations include the use of model hosts, STH species other than the target species, laboratory strain of the target STH species, unnatural infection and other limitations. With the use of model hosts such as golden hamsters, mice rats, and beagles, and laboratory STH species/strains that are genetically different from natural species/strains, it is unclear if any of the vaccines will translate to humans, and such studies are warranted. Also, the use of unnatural infections such as the diffusion chambers for *S. stercoralis* in mice, or inoculating mice with ascarids, which are not able to establish patent infections in the mouse small intestine, add further limitation for extrapolating to humans. Furthermore, most of the vaccine trails for each STH were conducted by only one or two different labs. More technical, laboratory-to-laboratory variations such as different laboratory STH strains, different viability and dose of inocula, different host species or strains and just simply different reagents, equipment and researchers could cause extensive variation in results. As the field of STH vaccinology continues to grow, the reproducibility of the results obtained for these recombinant subunit vaccines will likely be addressed.

### How can we better streamline antigen selection?

If blocking/neutralizing antibodies really are the bases of the protection achieved from the previous studies on recombinant subunit vaccines for STHs, then the antigens must have essential/important functions for infection. With the advent of whole genome sequencing combined with transcriptomics and proteomics, we are now capable of filtering out candidate antigens unlikely to be important for infection and/or to be present in immunologically inaccessible sites within the worms through bioinformatics and computational biology. Although a challenge, we can similarly prioritize for candidate antigens using the same methods, which will greatly increase the probability of testing antigens that have essential/important functions. The genomes have already been published for many of the most critical STHs (Table 1), and thus, much of the data needed for prioritization is already at hand.

Even with genomics-assisted selection of candidate antigens for experimental vaccination, importance/essentiality for infection can only be validated through reverse genetics. RNAi was used successfully to validate important functions of multiple *A. suum* antigens and subsequent vaccine studies resulted in comparatively high levels of protection (Islam *et al.* 2005*a*, *b*, Chen *et al.* 2011, 2012). Although RNAi has had variable success so far in other STHs such as *H. contortus* (Britton *et al.* 2012), this tool may still be useful for validation studies of particular antigens. An alternative reverse genetics tool for validation studies might be the CRISPR-Cas9 system for generating single gene knockouts. But although heritable loss-of-function mutants have been obtained in *C. elegans* using CRISPR-Cas9 (Friedland *et al.* 2013), no results have been published in STHs, so the efficacy of this system for validating important functions of candidate antigens is unknown at this time.

Another possible method to better streamline antigen selection could be to screen STH antigens for recognition by putative immune sera collected from people living in endemic regions that have high antibody titre and low worm burdens. However, if these antigens happen to be recognized by IgE in these humans, it is not likely that they will be useful as vaccine candidates given the findings with hookworm ASP-2 (Diemert *et al.* 2012).

### Are pan-anthelmintic vaccines possible?

The idea of a single, multivalent, pan-anthelmintic vaccine to control the three major human STHs (hookworms, *A. lumbricoides* and *T. trichiura*) has been proposed (Zhan *et al.* 2014). By including one or two recombinant protein immunogens for each STH, it is believed that all can be immunologically controlled simultaneously (Zhan *et al.* 2014). However, though such a vaccine would be ideal, the different locations that these STHs occupy within the GI tract and/or different feeding habits (Table 1) may present an enormous obstacle for a single vaccine. As mentioned above, adjuvants that worked well against one STH completely failed against another, and targeting hidden intestinal antigens in hookworms may work differently (or not at all) in *A. lumbricoides* and *T. trichiura*. And in combination with these three different STHs being phylogenetically distant relatives (i.e. from Nematoda clades V, III and I, respectively), the immunobiological factors involved in vaccine-induced protection against each of them is likely to be considerably different. On the other hand, pan-strongylid, pan-ascarid and pan-trichurid vaccines would seem to be more attainable goals given the appreciation of their phylogenetic relationships, and thus, biological similarities, and would also aim to target STHs of livestock. The latter ‘pan’ approach may also be achievable with mono or at most bivalent vaccines as antigens that are highly conserved within each group of target STHs, antigen(s) likely to have very similar and important functions for all of them, could be targeted.

### Conclusions/future directions

Recombinant subunit vaccines not only offer the potential to immunologically control STHs, but also are capable of being manufactured on a large enough scale to vaccinate over one fourth of all human beings and the majority of livestock in developing regions. Studies in hookworms have reported several recombinant immunogens with significant immunogenicity and protection, some of which progressed to clinical trials. The current best candidates in hookworms are those present in the adult intestine with roles in digestion of haemoglobin; those expressed in infective L3 are at risk of inducing allergic reactions in individuals with pre-existing IgE, and thus, should be avoided. Studies of another family of strongylids, the strongyloids, have revealed a number of immunogenic and protective recombinant immunogens in experimental vaccinations. But so far in the trichostrongyloids of livestock, minimal success has been achieved, likely due to the more technical factors of recombinant protein expression and/or purification. There are a number of recombinant immunogens from the ascarids that provided significant protection, some of which have been validated *via* RNAi to have important roles during infection. However, all of the target ascarid antigens are naturally immunodominant, abundant L3 surface proteins and thus, the studies in hookworms suggest potential issues for clinical application of the candidate immunogens. A few protective immunogens have been reported from *T. spiralis* that may also be effective against the relatively closely related trichurids.

Although there have been many successes with experimental vaccination using recombinant subunit vaccines, we still are far from understanding the mechanisms of vaccine-induced protection, which will require a sturdy bridge between vaccinology and immunobiology. A better understanding of the immunobiology and mechanism(s) of protection will lead to more potent, broad-spectrum vaccines. Also, more technical factors need to be better explored, such as why certain recombinant expression systems work and others do not for particular immunogens, how many injections and what doses per injection are optimal, which route of immunization is optimal (i.e. parenteral or oral/intranasal) and how long the immunological memory responses remain protective. Furthermore, many of the pre-clinical vaccine studies mentioned in this review focused on laboratory models, and thus, it is difficult to extrapolate to the target animals (and humans) as it is unclear whether the variation in immunological and biological factors will result in different vaccine efficacies. Better understanding these more technical factors and integrating genomics for testing higher priority target antigens will undoubtedly result in durable vaccines to protect our planet from these critical parasites.
